# Molecular Modulator Approach for Controlling the Length of Chiral 1D Single-Helical Gold Nanoparticle Superstructures

**DOI:** 10.1021/acs.chemmater.3c00590

**Published:** 2023-06-16

**Authors:** Yuyu Zhang, Sydney C. Brooks, Nathaniel L. Rosi

**Affiliations:** † Department of Chemistry, University of Pittsburgh, Pittsburgh, Pennsylvania 15260, United States; ‡ Department of Chemical and Petroleum Engineering, University of Pittsburgh, Pittsburgh, Pennsylvania 15260, United States

## Abstract

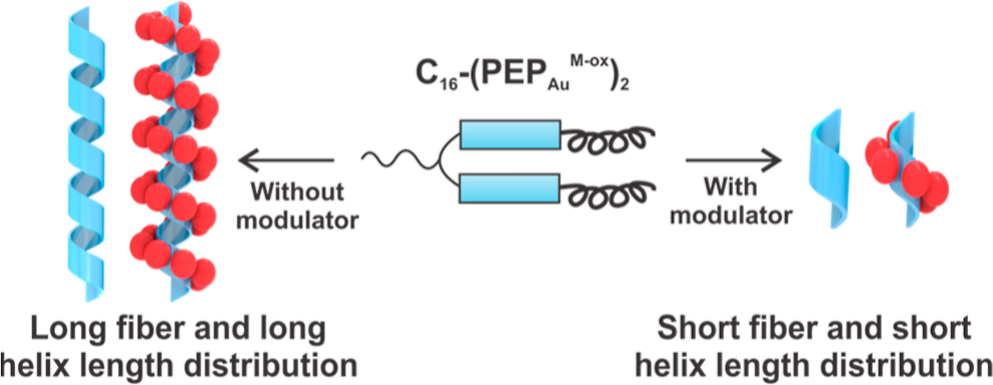

Peptide-based methods have proven useful for constructing helical gold nanoparticle superstructures that exhibit strong plasmonic chiroptical activity. Superstructure syntheses using the amphiphilic peptide conjugate C_16_-(AYSSGAPPM^ox^PPF)_2_ typically yield 1D helices with a broad length distribution. In this study, we introduce a molecular modulator approach for controlling helix length. It represents a first step toward achieving narrowly disperse populations of single helices fabricated using peptide-based methods. Varying amounts of modulator, C_16_-(AYSSGA)_2_, were added to C_16_-(AYSSGAPPM^ox^PPF)_2_-based single-helix syntheses, resulting in decreased helix length and narrowing of the helix length distribution. Kinetic studies of fiber assembly were performed to investigate the mechanism by which the modulator affects helix length. It was found that the modulator leads to rapid peptide conjugate nucleation and fiber growth, which in turn results in large amounts of short fibers that serve as the underlying scaffold for the single-helix superstructures. These results constitute important advances toward generating monodisperse samples of plasmonic helical colloids.

## Introduction

1D nanoparticle (NP) assemblies have attracted broad interest due to proposed applications that derive from their ensemble plasmonic properties and their ability to directionally transport light and electrons.^1−3^ The properties of these assemblies depend on factors such as NP size, shape, composition, interparticle distances, and also the 3D organization of component NPs along the axis of the 1D assembly. For example, 1D helical NP assemblies^4^ give rise to unique plasmonic chiroptical behavior which renders them potentially promising components of a variety of optical metamaterials.^5,6^ From a practical point of view, it is important to be able to control the length of these superstructures. For example, those with short, well-defined lengths may experience reduced losses during directional light or electron transfer. Furthermore, fabrication of monodisperse 1D superstructures may facilitate their processing and integration into other materials and devices.

Most 1D NP superstructures are prepared using template-based approaches.^1−4,7−9^ A wide variety of templates have been explored, from microorganisms to polymers to supramolecular fibers. In the latter case, molecular building blocks assemble to form 1D fibers which serve as scaffolds for organizing NPs. A distinct advantage of this approach is its molecular tunability, allowing for molecular-level control of the structure and properties of the NP superstructure. As an example, we have developed programmable peptide-based methods for assembling a wide variety of NP superstructures, including spherical architecture,^10−12^ 1D chains,^13,14^ and a diverse collection of chiral 1D helices.^15−21^ The basis of the methodology is peptide conjugate molecules that both bind to NPs and direct their assembly. They are designed to assemble into a soft template that serves as the underlying scaffold for arranging NPs. We have demonstrated how molecular modifications to the peptide conjugate translate into discernible and consequential changes in the morphology, structural metrics, and collective plasmonic properties of the NP superstructure.^11,17,18,21−24^ In the case of chiral helical gold (Au) NP superstructures, we have shown how the composition of the peptide conjugate can be tuned to control the degree of helicity (*e.g.*, double or single),^15,19^ helical pitch,^18,22^ particle dimensions,^23^ and helix handedness (left or right).^17^ However, we have yet to achieve helix length control. Our reported syntheses typically yield long helices (>1 μm) with broad length distributions (Figures S3 and S4). Helix length is an important parameter to control, especially when considering potential downstream applications that may require monodisperse populations of helices of a specific length. To address this challenge, we present here a molecular modulator approach for preparing Au NP single helices with controllable length and narrow length distributions. We show that superstructure length can be tuned by adjusting the amount of the molecular modulator added to the syntheses. Importantly, the resultant relatively monodisperse samples of Au NP single helices maintain strong plasmonic chiroptical activity.

## Results and Discussion

Controlling the length of 1D NP superstructures fabricated using soft template approaches requires control over the length of the template itself. Both physical and chemical approaches have been used to control template length. Physical approaches include ultrasonication^25−27^ and extrusion,^28^ but they are not particularly well-suited for controlling the length of an NP superstructure because they may disrupt or destroy the organization of NPs. Chemical approaches commonly involve the design of molecular additives that act as (i) “caps” to halt polymerization;^29−32^ (ii) “initiators” for controlling fiber nucleation;^33−36^ or (iii) agents that disassemble the preassembled template.^37,38^ Motivated to control the length of our single-helical Au NP superstructures, we drew inspiration from these prior studies and set forth to design peptide-based molecular modulators for controlling the length of the peptide fibers underlying the Au NP single helices.

**Figure 1:**
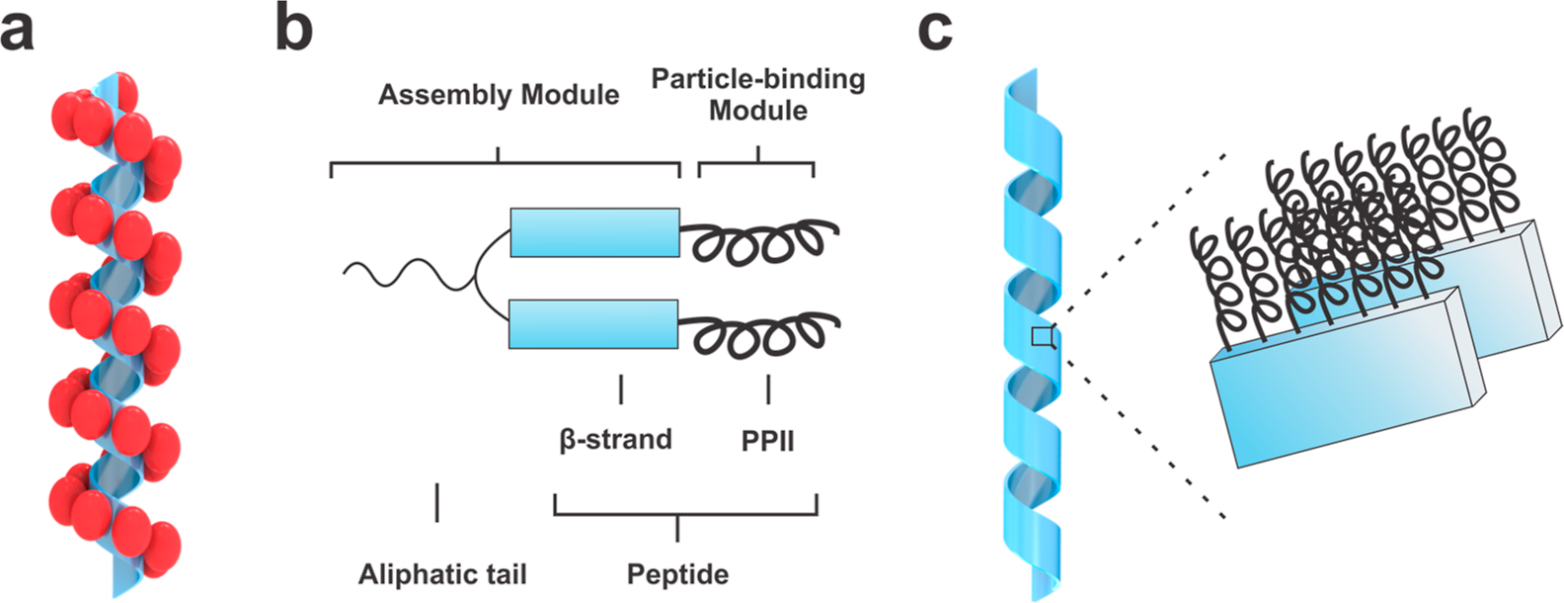
Assembly of Au NP single helices. (a) Cartoon representation of single helices in which the Au NPs decorate the external face of helical ribbons assembled from C_*x*_-(PEP_Au_^M-ox^)_2_. (b) C_*x*_-(PEP_Au_^M-ox^)_2_ conjugate (*x* = 16–22, PEP_Au_^M-ox^ = AYSSGAPPM^ox^PPF) consists of two modules: C_*x*_-AYSSGA is the assembly module and the C-terminus, PPM^ox^PPF, is the particle-binding module. (c) Helical ribbon assembly model: monolayers of C_*x*_-(PEP_Au_^M-ox^)_2_ arrange orthogonal to the ribbon surface. The ribbon thickness is approximately equal to the extended length of the peptide. The hydrogen bonding between the β-strands is along the long axis of the ribbons and intersheet stacking occurs along the axis perpendicular. The aliphatic tails (not shown) likely aggregate on the inner surface of the ribbon.

Our single-helical Au NP superstructures (Figure 1a) are prepared using a ‘divalent’ peptide conjugate consisting of two PEP_Au_^M-ox^ (AYSSGAPPM^ox^PPF; M^ox^ indicates methionine sulfoxide) head groups attached at their *N*-termini to an aliphatic tail (*e.g.*, C_16_–C_22_) (Figure 1b).^19,22^ These peptide conjugates assemble into 1D helical ribbons in aqueous buffers (Figure 1c). Through various microscopy, spectroscopy, and diffraction studies, we determined that these helical ribbons consist of a monolayer of C_*x*_-(PEP_Au_^M-ox^)_2_ arranged orthogonally to their surface (Figure 1c).^19^ C_*x*_-(PEP_Au_^M-ox^)_2_ can be divided into an “assembly module” and a “particle-binding module” (Figure 1b).^21^ Within the context of the 1D helical ribbon fibers, the C-terminus of PEP_Au_^M-ox^, PPM^ox^PPF, is exposed on the outer surface of the ribbon, exhibits PPII secondary structure, and serves as the “particle-binding module”. The *N*-terminal amino acids, AYSSGA, coupled with the aliphatic tail comprise the “assembly module”: AYSSGA engages in β-sheet formation and the aliphatic tails promote aggregation in aqueous media. This assembly serves as a basis for designing a molecular modulator for controlling fiber length.

Using single helices prepared with C_16_-(PEP_Au_^M-ox^)_2_ (Figure S1a,b) as the basis for this study, we designed a modulator, C_16_-(AYSSGA)_2_ (Figures S1b and S2b), which is sequence-matched to the C_16_-(PEP_Au_^M-ox^)_2_ β-sheet region and contains only its “assembly module” components. Based on our assembly model, we postulated that C_16_-(AYSSGA)_2_ would readily form fibers but would not bind to Au NPs or direct their assembly because it lacks the particle-binding module. We designed a set of experiments in which incremental amounts of the modulator were added to single-helix syntheses to examine the effect of the modulator on helix length. The single-helix products were analyzed using transmission electron microscopy (TEM). In brief, each synthesis was repeated to ensure reproducibility and at least 30 TEM images of product were collected for each synthetic replicate. The lengths of the helices within these images were measured, and the average helix length and helix length distribution were calculated for each synthetic condition.

We first prepared Au NP single helices using C_16_-(PEP_Au_^M-ox^)_2_ by following our previously reported synthetic procedure (Figure S3).^19^ Based on measuring 260 helical superstructures, helix lengths ranged from ∼80 to 25,000 nm, with a median length of 693 nm and an average length of ∼1740 nm. ∼40% of the helices were longer than 1000 nm and ∼3% were longer than 10,000 nm (Figure S4). We concluded from these results that unmodulated syntheses yield a broad distribution of helix lengths.

We next studied the assembly of C_16_-(AYSSGA)_2_ and determined whether it could direct NP assembly. C_16_-(AYSSGA)_2_ was incubated in aqueous buffer overnight, and the resulting assemblies were then imaged using TEM. Negatively stained TEM images revealed a high density of short fibers, most with lengths less than 1 μm (Figure S6a,b); for comparison, C_16_-(PEP_Au_^M-ox^)_2_ typically assembles into much longer fibers (>5 μm) under the same conditions (Figure S5). Circular dichroism (CD) and Fourier transform infrared (FTIR) spectroscopy studies were conducted to investigate the molecular structure of these fibers. The CD spectrum of the modulator fibers exhibited a slightly positive peak at ∼198 nm and a negative peak at ∼215 nm (Figure S6c) and the amide I stretch in the FTIR spectrum appeared at 1634 cm^–1^ (Figure S6d), which are consistent with β-sheet secondary structure.^39−42^ In addition, a peak at ∼2942 cm^–1^ in the FTIR spectrum corresponding to C–H stretches indicates the ordered packing of the aliphatic tails.^43^ When C_16_-(AYSSGA)_2_ was incubated in Au NP helix synthesis and assembly conditions,^19^ only discrete, nonassembled, Au NPs were observed (Figure S6e,f). These data confirm that modulator C_16_-(AYSSGA)_2_ assembles into fibers which do not direct the assembly of Au NPs. Notably, the high density of short fibers suggests rapid fiber nucleation and growth, indicating that the modulator has a high propensity to assemble into fibers under the conditions studied.

**Figure 2:**
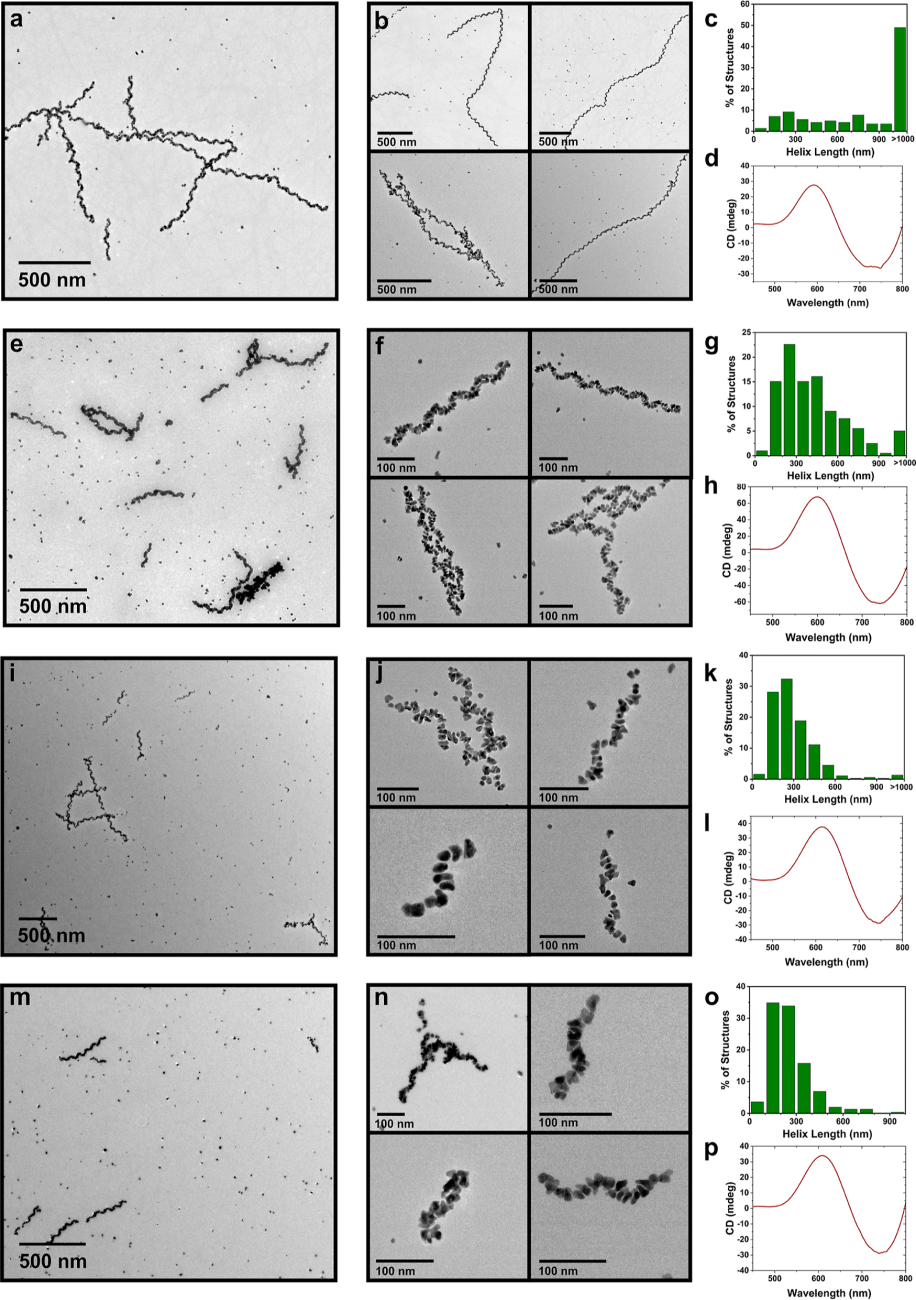
TEM images of Au NP single helices synthesized from C_16_-(PEP_Au_^M-ox^)_2_: C_16_- (AYSSGA)_2_ at mole ratios of (a,b) 10:1, (e,f) 10:5, (i,j) 10:10, and (m,n) 10:15. Helix length distribution of (c) 10:1, (g) 10:5, (k) 10:10, and (o) 10:15; CD spectra of single helices: (d) 10:1, (h) 10:5, (l) 10:10, and (p) 10:15.

We proceeded to explore how the addition of the modulator to single helix syntheses affects length distribution. The mole ratio of C_16_-(PEP_Au_^M-ox^)_2_ to the modulator, X/Y, was varied between 10:1 and 10:25, where X and Y indicate nanomoles of C_16_-(PEP_Au_^M-ox^)_2_ and C_16_-(AYSSGA)_2_, respectively, in the NP assembly reaction. The effect of the modulator on single helix length is summarized in Table 1 and Figure 2. When only 1 nmol of the modulator is added to a typical synthesis (10:1), the average helix length and distribution are similar to the control study (Figures 2a–c, S7, and S8). However, increased amounts of the modulator result in shorter helices and narrower helix length distribution. At ratios of 10:5 or higher, over 90% of the helices are shorter than 1000 nm. In general, the helix length distribution narrows as we increase the amount of the modulator. For 10:5 (Figures 2e–g, S9, and S10), only ∼5.0% of the helices are longer than 1000 nm, the median length is ∼385 nm, and the average length is ∼442 nm, a distinctly different distribution compared to C_16_-(PEP_Au_^M-ox^)_2_ alone. When we increase the amount of the modulator to 10 nmol (10:10), only 1.3% of the helices are longer than 1000 nm, and the median and average lengths decrease to ∼254 and ∼296 nm, respectively (Figures 2i–k, S11, and S12). At 10:15, when more modulators than C_16_-(PEP_Au_^M-ox^)_2_ is present in solution, all measured helices are shorter than 1000 nm, with a median length of ∼231 nm and an average length of ∼261 nm (Figures 2m–o and S13). When we increase the modulator ratio even further, 10:25 (Figures S14 and S15), all counted helices are less than 1000 nm with 98.1% shorter than 500 nm (Figure S16). The median and average lengths are ∼174 and ∼199 nm, respectively. Notably, short helices produced at ratios 10:5, 10:10, and 10:15 exhibit strong plasmonic chiroptical activity (Figures 2d,h,l,p, S17, and S18), confirming that helix length, provided it completes at least one turn of the helix, should not impact the plasmonic coupling and the intensity of the plasmonic chiroptical response.^5^ Helices formed from 10:25 did not show an observable plasmonic chiroptical response (Figure S19), perhaps due to the structural irregularity of the product (Figures S14 and S15). Further structural analysis of the helices indicates that increasing the amount of the modulator results in similar helical pitch length and, in general, similar NP dimensions (Table S1). We do note that the shortest helices produced from the 10:25 syntheses were less well-defined, and the NPs were more irregularly shaped; while we cannot definitively explain this phenomenon at this stage, we do comment on it further when we discuss the proposed mechanism of modulator-mediated length control (*vide infra*).

**Table 1:** Helix Length Data of Single Helices Prepared Using C_16_-(PEP_Au_^M-ox^)_2_ and Mixtures of C_16_-(PEP_Au_^M-ox^)_2_ and C_16_-(AYSSGA)_2_ (Modulator)

mole ratio (nmol:nmol)		helix length			
C_16_-(PEP_Au_^M-ox^)_2_	modulator	>1000 nm	<500 nm	median (nm)	average (nm)
10	0^a^	∼39.6%	∼42.3%	∼634	∼1740
10	1^b^	∼49.5%	∼27.3%	∼963	∼1306
10	5^c^	∼5.0%	∼80.8%	∼385	∼442
10	10^d^	∼1.3%	∼92.0%	∼254	∼296
10	15^e^	∼0%	∼95.1%	∼231	∼291
10	25^f^	∼0%	∼98.1%	∼174	∼199
10	10	N/A	N/A	N/A	N/A

a Measurement based on 260 structures.

b Measurement based on 143 structures.

c Measurement based on 199 structures.

d Measurement based on 377 structures.

e Measurement based on 304 structures.

f Measurement based on 214 structures.

We also analyzed how the modulator affects fiber length. A typical Au NP assembly experiment yields both Au NP single helices as well as undecorated peptide fibers. To visualize these fibers, we negatively stained the TEM grids for samples 10:1, 10:5, 10:10, and 10:25, collected TEM images (Figures S8, S10, S12, and S15), and then measured the fiber lengths. We found that fiber length distribution for each sample mirrored helix length distribution: an increasing amount of the modulator resulted in shorter fiber length distribution (Figure S20 and Table 2). Fiber lengths are generally longer than 3000 nm for 10:1 (Figure S7). For 10:5, the median length is ∼1005 nm, the average length is ∼1093 nm, and ∼50% of the fibers are shorter than 1000 nm (Figure S20). 10:5 exhibits shorter fiber length distribution than 10:1. With increasing amounts of the modulator, the fiber lengths decrease. At 10:10, the average and median lengths are ∼653 nm and ∼547 nm, respectively, and ∼80% of the fibers are shorter than 1000 nm (Figure S20 and Table 2). Most of the counted fibers were shorter than 1000 nm for 10:25, with an average length of ∼348 nm and a median of ∼311 nm (Figure S20 and Table 2). Thus, the addition of the modulator results in shorter fiber lengths and length distributions overall.

**Table 2:** Fiber Length Data for C_16_-(PEP_Au_^M-ox^)_2_ and Mixtures of C_16_-(PEP_Au_^M-ox^)_2_ and C_16_- (AYSSGA)_2_ (Modulator) (Measurement Based on 150 Counts)

mole ratio (nmol:nmol)		fiber length		
C_16_-(PEP_Au_^M-ox^)_2_	modulator	>1000 nm	median (nm)	average (nm)
10	1	∼100%	—	—
10	5	∼50%	∼1005	∼1093
10	10	∼19.5%	∼547	∼653
10	25	∼1%	∼311	∼348

As a first step toward understanding the modulator’s role in reducing helix and fiber lengths, we conducted spectroscopic studies to determine whether it affects the molecular structure of the fibers. Fiber samples were prepared by incubating C_16_-(PEP_Au_^M-ox^)_2_ with the modulator overnight in aqueous 4-(2-hydroxyethyl)-1-piperazineethanesulfonic acid (HEPES) buffer at the ratios listed in Table 1. CD studies indicate that all samples have positive peaks at wavelength <200 nm and negative peaks between ∼211 nm and 215 nm, indicating β-sheet secondary structure (Figure S21).^39,40^ The strength of the signal generally correlates with the total concentration of the peptide conjugate in the different samples. In addition, the FTIR spectra display an amide I band at ∼1630 cm^–1^ and a C–H stretch at ∼2920 cm^–1^, which are characteristic of β-sheet secondary structure^41,42^ and ordered packing of the hydrophobic tails,^43^ respectively (Figure S22). These spectral data are consistent with what we have previously reported for C_16_-(PEP_Au_^M-ox^)_2_ fibers,^22^ suggesting that the modulator does not disrupt the fibers’ molecular structure.

We considered two possible pathways by which the molecular modulator might affect fiber growth and length as well as the length of the single-helical Au NP superstructures: (i) molecular “capping” of fibers (Pathway 1) and (ii) initiation of fiber nucleation (Pathway 2). The “capping” mechanism assumes that the modulator would competitively add to the growing ends of C_16_-(PEP_Au_^M-ox^)_2_-based fibers and halt their growth; furthermore, it assumes that either C_16_-(PEP_Au_^M-ox^)_2_ nucleation and growth precedes the modulator nucleation and growth or that they have similar assembly kinetics. The second proposed mechanism considers the strong assembly propensity of the modulator due to its hydrophobic to hydrophilic ratio, and its ability to promote rapid formation of nucleation seeds onto which C_16_-(PEP_Au_^M-ox^)_2_ may associate and then grow into fibers. Thus, the modulator would accelerate C_16_-(PEP_Au_^M-ox^)_2_ fiber growth, resulting in short fiber and helix length distribution.

**Figure 3:**
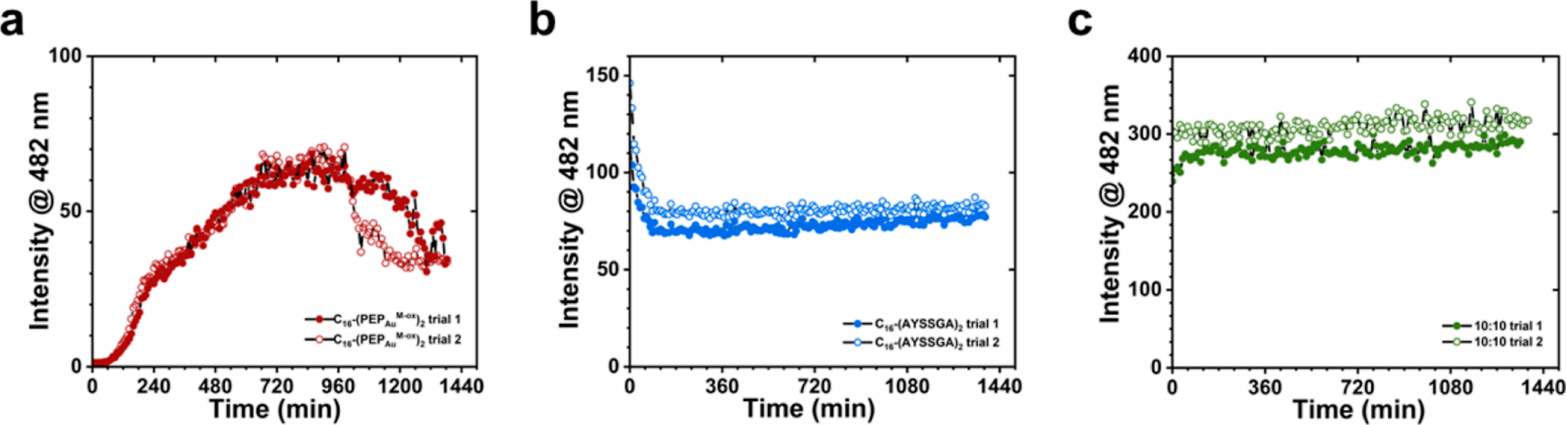
ThT fluorescence monitoring of the assembly of (a) two replicates of C_16_-(PEP_Au_^M-ox^)_2_, (b) two replicates of C_16_-(AYSSGA)_2_, and (c) two replicates of 10:10 C_16_-(PEP_Au_^M-ox^)_2_: C_16_-(AYSSGA)_2_.

To distinguish between these two proposed pathways, we conducted Thioflavin T (ThT) fluorescence assays to examine how the modulator influences fiber growth kinetics,^44,45^ reasoning that Pathway 2, in which the modulator is postulated to rapidly nucleate to yield “nucleation seeds” for fiber growth, would be supported by observation of rapid growth kinetics and large populations of shorter fibers. The ThT fluorescence profile for C_16_-(PEP_Au_^M-ox^)_2_ assembly exhibits a sigmoidal curve consisting of a lag phase (0–60 min), an elongation phase (60–660 min), and an equilibrium phase (660–1380 min) (Figure 3a), which suggests that C_16_-(PEP_Au_^M-ox^)_2_ undergoes cooperative supramolecular polymerization.^46^ In comparison, the modulator alone exhibits a dramatically different fluorescence profile, with an immediate equilibrium stage and without observable nucleation and elongation stages (Figure 3b). The apparent instantaneous assembly in aqueous buffer can be understood when considering that the modulator, C_16_-(AYSSGA)_2_, contains only the “assembly” components of C_16_-(PEP_Au_^M-ox^)_2_ and has a high hydrophobic to hydrophilic ratio. However, the modulator does exhibit supramolecular cooperative assembly consisting of (i) nucleation stage (0–60 min), (ii) elongation stage (60–90 min), and (iii) equilibrium stage (90 min and beyond) when its assembly is monitored in a more hydrophobic solution environment, 1:1 acetonitrile/H_2_O (Figure S23).

We collected TEM images at different timepoints along the fiber assembly profile to track fiber growth and the evolution of fiber length. For C_16_-(PEP_Au_^M-ox^)_2_, at *t* = 0, which corresponds to the onset of the nucleation phase, we observed little fiber formation (Figure S24a). During the fiber growth phase, at *t* = 1, 2, and 3 h, we observed long fibers (>3 μm) and increasing fiber density on the TEM grids (Figure S24b–d). At 18 h, during the equilibrium phase, we again observe a dense network of long fibers (Figure S24e). In the case of the modulator, TEM images collected at *t* = 0 show a high density of short fibers (Figure S25a), which would be expected if there were an initial burst of nucleation sites followed by fiber growth. Modulator fibers are significantly shorter than C_16_-(PEP_Au_^M-ox^)_2_ fibers, with an average and median length of ∼485 and ∼400 nm, respectively, at 1 h and ∼999 and ∼910 nm at 3 h (Table S2).

The ThT fluorescence profile of the 10:10 mixture of C_16_-(PEP_Au_^M-ox^)_2_ and modulator indicates immediate nucleation followed by elongation (0–1 h) and then equilibrium (1 h and beyond) (Figure 3c). TEM fiber length monitoring of this sample reveals a high density of short fibers (median and average fiber length <600 nm) at *t* = 0 and 30 min (Figure S26a,b and Table S3). At *t* = 4 h, we observed a slight increase in fiber length, with average and median of ∼926 and ∼720 nm, respectively (Table S3 and Figure S26e). Overall, the 10:10 sample exhibits a significantly shorter fiber length distribution and a much more rapid assembly profile compared to C_16_-(PEP_Au_^M-ox^)_2_ alone. To further examine the influence of the modulator, we conducted the ThT fluorescence assay for a 10:20 sample (Figure S27). Its assembly profile is similar to that observed for 10:10 with immediate nucleation, followed by elongation (0–300 min), and lastly equilibrium (300–1380 min). 10:20 exhibits shorter fibers compared to 10:10 and pure C_16_-(PEP_Au_^M-ox^)_2_, with median and average fiber lengths of <500 nm at *t* = 0 min, 1, 3, and 48 h (Figure S28 and Table S4).

From these mechanistic studies, we conclude that the modulator, a β-sheet assembly agent, nucleates rapidly, yielding numerous seeds to which free conjugates can add to form fibers. This results in a large population of short fibers (Pathway 2). When the modulator is added to C_16_-(PEP_Au_^M-ox^)_2_, it promotes rapid nucleation and growth; the modulator nuclei can serve as seeds for growth of both C_16_-(PEP_Au_^M-ox^)_2_ and modulator fibers and potentially fibers containing a mixture of the modulator and C_16_-(PEP_Au_^M-ox^)_2_. Since C_16_-(PEP_Au_^M-ox^)_2_ contains the same assembly module as the modulator, it is reasonable to assume that monomers of C_16_-(PEP_Au_^M-ox^)_2_ could add to the modulator nuclei. We postulate that syntheses containing a larger amount of the modulator (*e.g.*, 10:25) yield fibers constructed from a significant amount of the modulator. Since the modulator does not contain the particle-binding module which helps cap particle growth, the helices that form on these fibers are composed of more irregularly shaped particles (*vide supra*).

## Conclusions

We established that a molecular modulator approach can be used to control amphiphilic peptide conjugate fiber growth profiles and can be leveraged to control the length of fibers and helical Au NP assemblies. The introduction of modulator C_16_-(AYSSGA)_2_ to the C_16_-(PEP_Au_^M-ox^)_2_ Au NP assembly system significantly affects the length of single-helical superstructures: as the amount of the modulator increases with respect to a fixed concentration of C_16_-(PEP_Au_^M-ox^)_2_, peptide conjugate fiber length and Au NP superstructure length decrease. ThT fluorescence kinetic studies and fibril length evolution imaging provided mechanistic insights which suggest that modulator accelerates the nucleation kinetics of the entire assembly system, leading to overall shorter fiber lengths. We used this molecular modulator approach to achieve single-helix samples with average and median helix lengths between ∼200–500 and ∼200–400 nm, respectively. Significantly, these samples maintained the intense plasmonic chiroptical response which has previously been observed for samples generated from unmodulated syntheses.

## Materials and Methods

### Materials

All chemicals were purchased from commercial sources and used without purification unless otherwise noted. Fmoc-protected amino acids (Fmoc = fluorenylmethyloxycarbonyl), Fmoc-protected Phe and Ala NovaSynTGA resins, and O-(1H-6-Chloro-benzotriazole-1-yl)-1,1,3,3-tetramethyluronium hexafluorophosphate (HCTU) were purchased from Novabiochem; dialysis mini tubes (D-tube) were purchased from EMD Millipore; *N*,*N*-dimethylformamide (DMF), methylene chloride (DCM), and 1.0 M HEPES) buffer (pH = 7.3 ± 0.1) were purchased from Fisher Chemical; diethyl ether (Et_2_O, 99.5%, extra dry, stabilized, AcroSeal), trifluoroacetic acid (TFA, 99% extra pure), and ThT dye were purchased from Acros Organics; 5-azidovaleric acid was purchased from TCI Chemical; formic acid (88%, Baker analyzed) was purchased from JT Baker. Carbon support film (5–6 nm thick, 400 mesh) copper grids were purchased from Electron Microscopy Sciences. All other chemicals were purchased from Sigma-Aldrich. Nanopure water (18.1 mΩ, Barnstead Diamond purification system) was used for all aqueous studies.

### Reverse-Phase High-Pressure Liquid Chromatography Purification

All synthesized peptides and peptide conjugates were purified under ambient temperature using an Agilent 1200 liquid chromatographic system equipped with a diode array and multiple-wavelength detectors and using a Zorbax-300SB C_18_ column. A linear gradient of a binary solvent system (A: 0.1% formic acid in Nanopure water; B: 0.05% formic acid in acetonitrile) ramping from 95% buffer A and 5% buffer B to 5% buffer A and 95% buffer B over a period of 30 min was used to purify the peptides and peptide conjugates.

### UV–Vis Spectroscopy

All synthesized peptides and peptide conjugates were quantified based on the absorbance of tyrosine (1280 M^–1^ cm^–1^) at 280 nm. UV–vis absorption spectra were collected using an Agilent 8453 UV–vis spectrometer equipped with deuterium and tungsten lamps and using a quartz cuvette with 10 mm path length.

### Liquid Chromatography–Mass Spectroscopy

Liquid chromatography–mass spectrometry (LCMS) spectra were collected on a Shimadzu LCMS-2020 instrument using a direct injection method with an electron spray ionization (ESI) probe in positive and negative scan mode over a total run of 6 min.

### Fmoc Solid-Phase Peptide Synthesis

Peptides were synthesized by manual solid-phase peptide synthesis using a CEM MARS microwave (Mattews, NC, USA) and NovaSyn TGA Fmoc resin. The synthesis protocol consists of (i) resin preparation and deprotection, (ii) sequential amino acid coupling followed by Fmoc-deprotection, (iii) capping of 5-azido pentanoic acid to the *N*-terminus, and (iv) peptide cleavage from resin. To activate the amino acids, HCTU (5 equiv to resin) and *N*,*N*-diisopropylethylamine (7 equiv to resin) in 1-methyl-2-pyrrolidinone were added to Fmoc-protected amino acids (5 equiv to resin) and allowed to sit for 5–7 min. The coupling reaction occurred under 1 min ramp from room temperature to 75 °C, followed by a 5 min hold. The deprotection solutions consisted of 20% v/v 4-methylpiperidine in DMF, and the reaction proceeded under a 1 min ramp to 75 °C, followed by a 2 min hold. To cap the *N*-terminus, 5-azidovaleric acid (5 equiv to resin) was activated and coupled following the typical procedure (no final deprotection). The resin was then washed with DMF (3X), DCM (3X), methanol (3X) and then dried under vacuum for 30 min. To cleave the peptide, the resin was soaked in a peptide cleavage cocktail [90% TFA, 5% triisopropylsilane, and 5% Nanopure H_2_O] for 4 h. Cold Et_2_O was added to precipitate the peptide. After centrifugation, the supernatant was decanted and 1:1 acetonitrile(ACN):H_2_O was added to dissolve the pellet. The peptide solution was lyophilized using a Labconco Freeze-Dryer system (Kansas City, MO, USA), and the solid peptide was stored at −20 °C.

### Peptide Conjugate Synthesis

Detailed protocols can be found in previous reports.^18,19^ Briefly, oxidized N_3_-PEP_Au_^M-ox^ was prepared by adding 8 μL of 50% hydrogen peroxide (H_2_O_2_) solution to ∼5 mg of peptide dissolved in 1 mL of 1:1 ACN/H_2_O. C_16_-dialkyne was attached to each azido-terminated peptide sequence via copper-catalyzed cycloaddition to synthesize C_16_-(PEP_Au_^M-ox^)_2_ and modulator, C_16_-(AYSSGA)_2_.

### Fiber Assembly

250 μL of 0.1 M HEPES buffer was added to lyophilized peptide conjugates. The solution was sonicated for 5 min. 2.5 μL of 0.1 M calcium chloride (CaCl_2_) was then added to promote assembly.

### Au NP Helix Assembly

250 μL of 0.1 M HEPES buffer was added to lyophilized peptide conjugates. For short helix preparation, the peptide conjugates consisted of a mixture of C_16_-(PEP_Au_^M-ox^)_2_ and modulator [C_16_-(AYSSGA)_2_] at appropriate nanomole ratios. The solution was sonicated for 5 min, 2.5 μL of 0.1 M CaCl_2_ was added, and the solution was left undisturbed for 25 min. 1 μL of a gold precursor solution [1:1 1.0 M triethylammonium acetate buffer:0.1 M aqueous gold (III) chloride trihydrate (HAuCl_4_)] was added. A black precipitate forms after ∼2 s, at which time the solution was immediately vortexed for ∼30 s. The solution was allowed to sit undisturbed for 16 h before further characterization.

### CD Spectroscopy

CD studies were performed on an Olis DSM 17 CD spectrometer with a quartz cuvette (1 mm path length) at 25 °C with 8 nm/min scan rate and 2 nm bandwidth. For secondary structure studies, solid peptide conjugates were dissolved in 0.01 M HEPES and spectra were collected from 190 to 250 nm. For plasmonic studies, spectra were collected from 450 to 800 nm.

### Attenuated Total Reflectance Fourier Transform Infrared Spectroscopy

Attenuated total reflectance Fourier transform infrared (ATR-FTIR) spectra were collected on a PerkinElmer Spectrum 100 spectrometer (Waltham, MA, USA) equipped with a universal ATR sampling accessory. Spectra were collected between 450 and 4000 cm^–1^ and processed using PerkinElmer Spectrum Express software. Peptide conjugates were dissolved in 0.1 M HEPES for 1 day, and then, the fibers were dialyzed in Nanopure H_2_O using a dialyzer mini tube (Millipore, D-Tube^TM^, MWCO 12–14 kDa, catalog no. 71505–3). The samples were concentrated by air evaporation. 1 μL of the concentrated samples was drop-cast onto ATR crystal surface and allowed to air dry. The collected spectra were background-corrected in air.

### Transmission Electron Microscopy

C_16_-(PEP_Au_^M-ox^)_2_ and “10:10” negatively stained images were collected on an FEI Morgagni 268 instrument operated at 80 KV and equipped with an AMT side mount CCD camera system. The remaining TEM images were collected on a Hitachi H-9500 microscope (Chiyoda, Tokyo, Japan) equipped with a Gatan CCD camera analyzed by Digital Micrograph software operating at 300 KV. Previously reported sample preparation protocols were used.^19,22^ All TEM image measurements were analyzed using ImageJ (NIH, USA) software.^47,48^

### ThT Assays

ThT fluorescence assays were conducted in a 96-well flat bottom black plate (Greiner Bio-One, catalog no.655209) at 26 °C in a Tecan Infinite M1000 Pro fluorescence plate reader. Lyophilized peptide conjugates were dissolved in 250 μL of 5 μM ThT in 0.1 M HEPES buffer, and 2.5 μL of 0.1 M CaCl_2_ was added. After brief vortexing, the sample solution was transferred to each well. The ThT fluorescence kinetic profile was recorded with either 5 or 8 min reading intervals and 5 s shaking (3 mm in linear amplitude and 372–414 rpm in linear frequency) before each read (440 nm excitation and 482 nm emission). At least two replicates were collected for each assay. All fluorescence spectra signals were background corrected.

## Supporting Information Available

LC–MS, CD, FT-IR spectra, additional TEM images, structural parameter data, and length distribution data (PDF)
