# Supplementary material for: Molecular Modulator Approach for Controlling the Length of Chiral 1D Single-Helical Gold Nanoparticle Superstructures
Source: Chem Mater. 2023 Jun 16;35(13):5071–8. doi: 10.1021/acs.chemmater.3c00590 (PMC10339826; doi:10.1021/acs.chemmater.3c00590)
Supplement: Supplementary file 1 — cm3c00590_si_001.pdf [file cm3c00590_si_001.pdf]

Supporting Information

Molecular Modulator Approach for Controlling the Length of Chiral 1-D Single-Helical Gold Nanoparticle Superstructures

Yuyu Zhang[†], Sydney C. Brooks[†], Nathaniel L. Rosi*[†‡]

† Department of Chemistry, University of Pittsburgh, Pittsburgh, Pennsylvania 15260, United States

‡ Department of Chemical and Petroleum Engineering, University of Pittsburgh, Pennsylvania 15260, United States

a

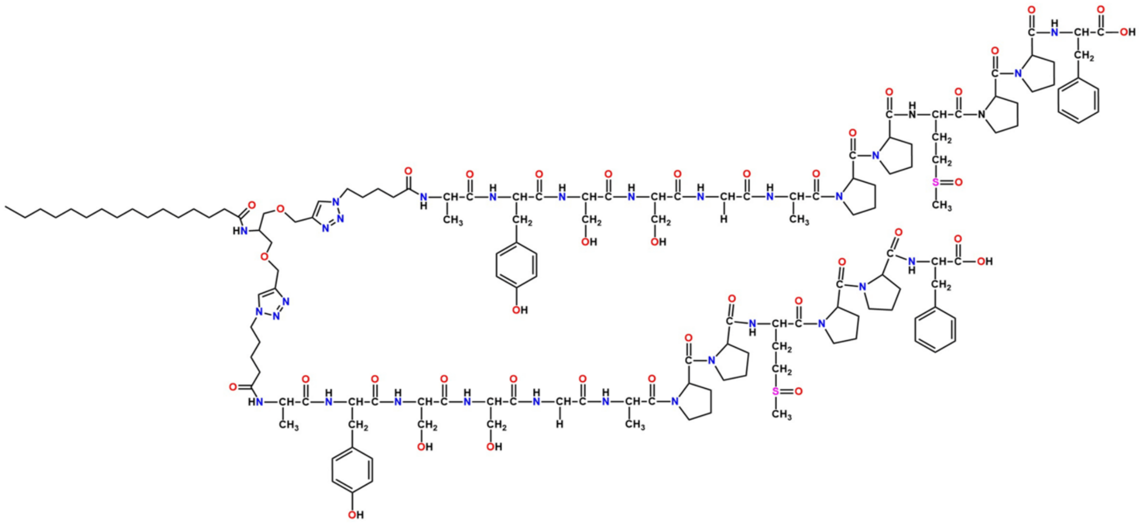

b

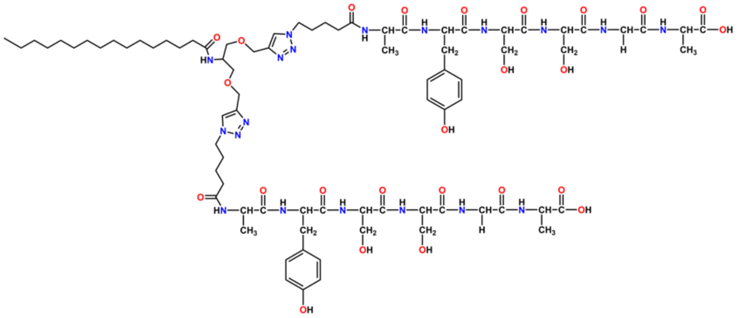

Figure S1. Chemical structures of (a) C_{16}-($PEP_{Au}^{M\text{-}ox}$)$_2$ and (b) C_{16}-(AYSSGA)$_2$.

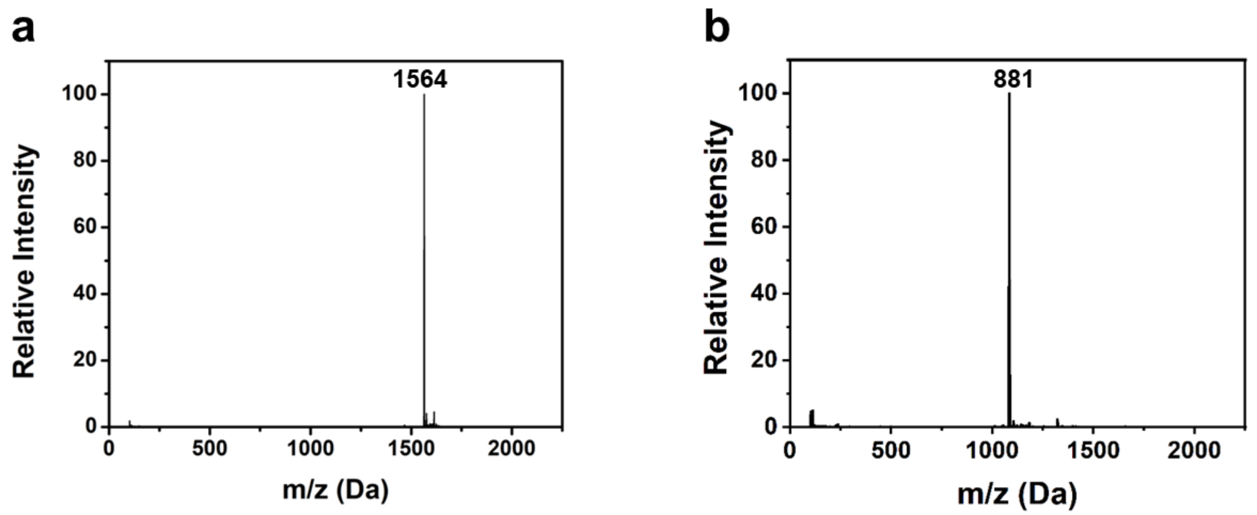

Figure S2. LC-MS spectra of (a) C_{16}-($PEP_{Au}^{M\text{-}ox}$)$_2$, m/z = 1564 Da (M-2H$^+$)/2 and (b) C_{16}-(AYSSGA)$_2$, m/z= 881 Da (M-2H$^+$)/2).

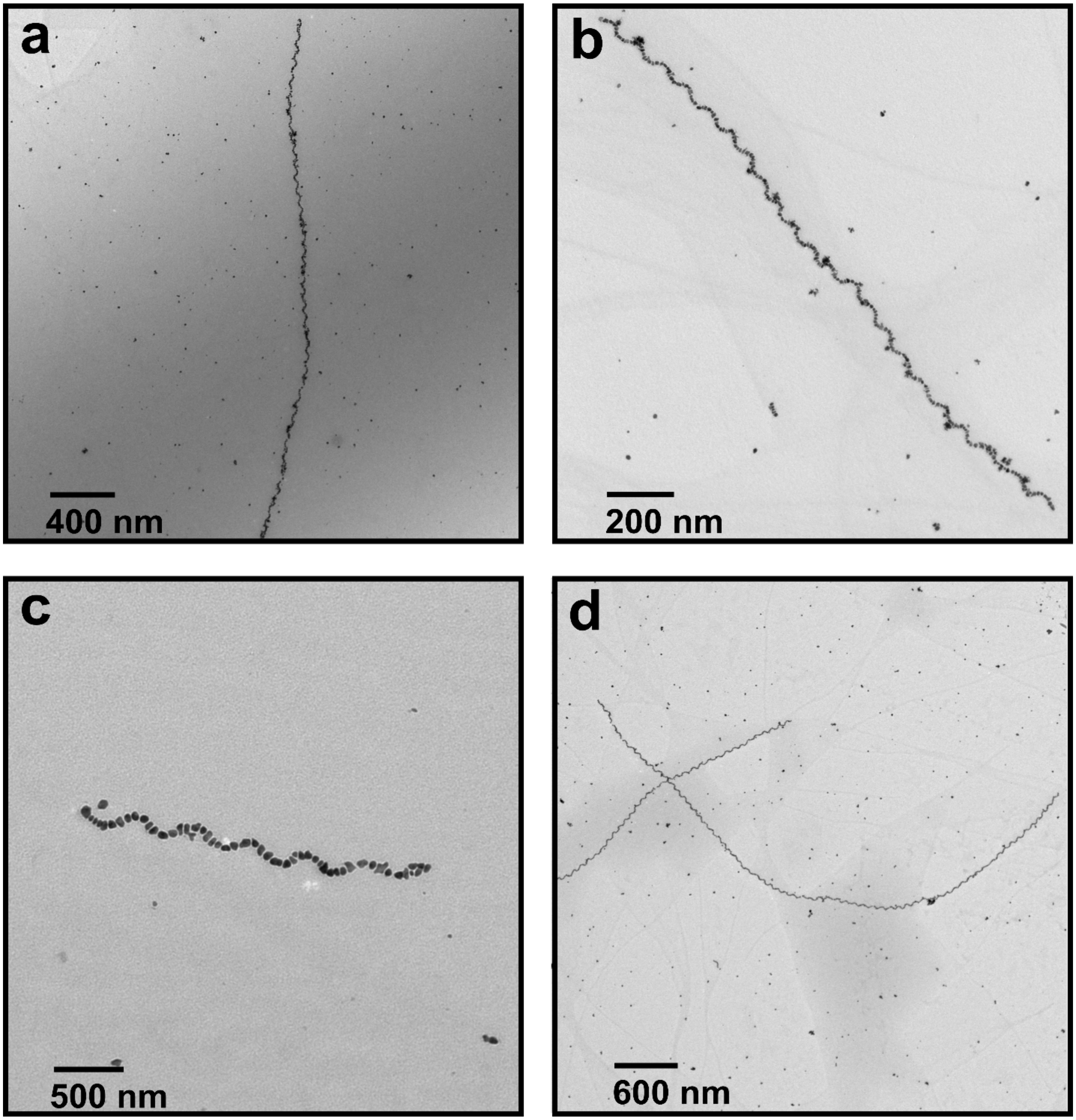

Figure S3. Representative TEM images of single helices fabricated using C_{16}-($PEP_{Au}^{M\text{-}ox}$)$_2$. These images, and others, were used for determining the helix length distribution.

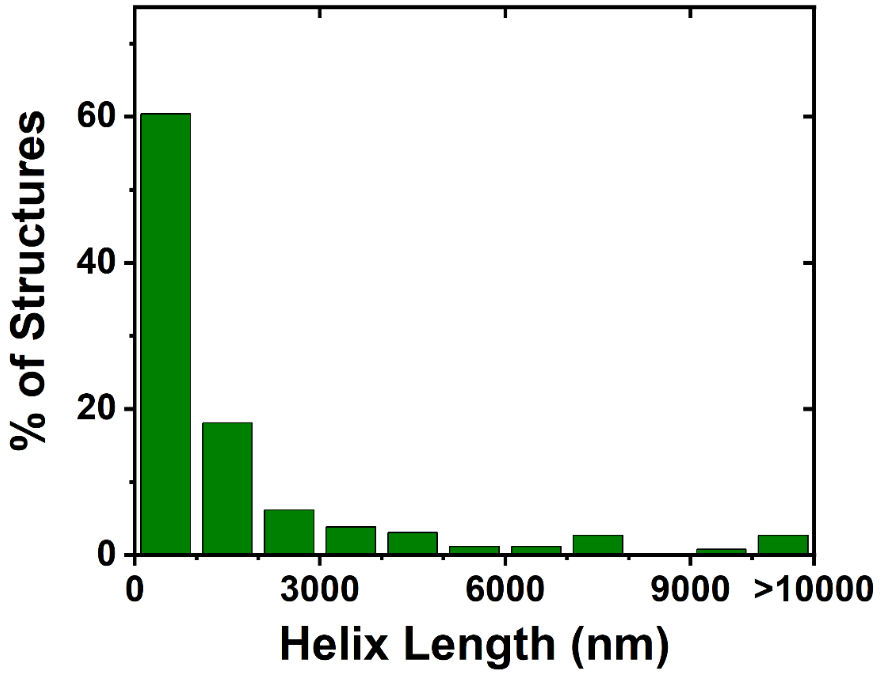

Figure S4. Helix length distribution for single helices prepared using C_{16}-($PEP_{Au}^{M\text{-}ox}$)$_2$. Helix lengths ranged from ~80-25000 nm with an average of ~1740 nm and median of 693 nm. 39.6% superstructures are longer than 1000 nm. Measurement is based on 260 counts.

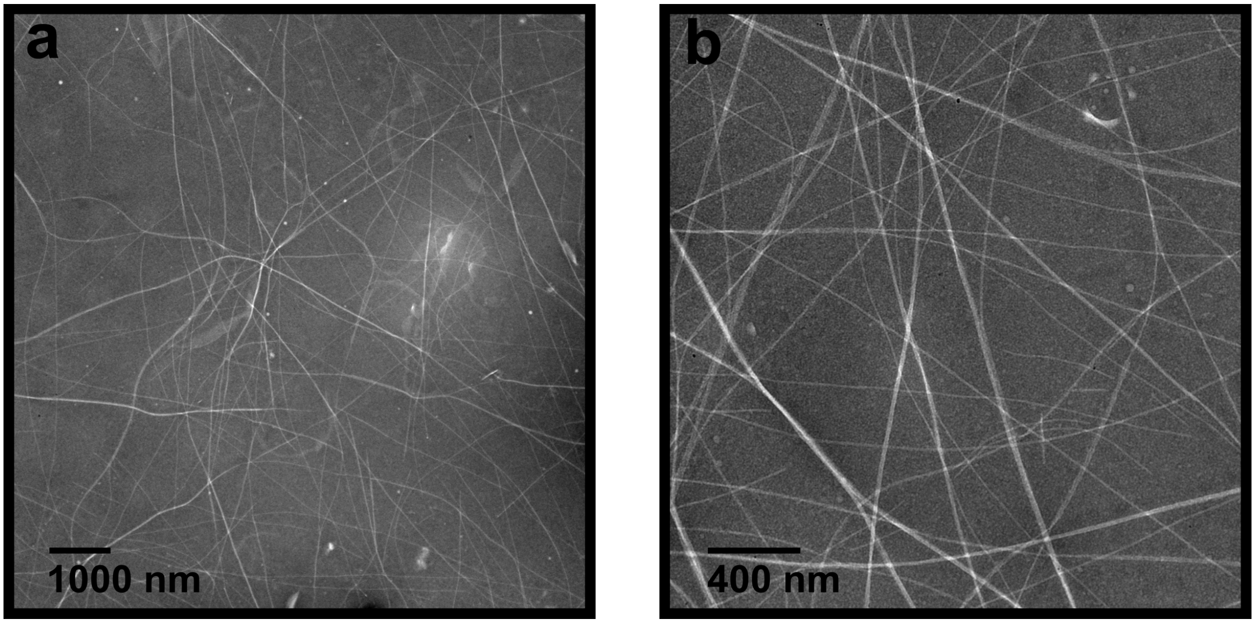

Figure S5. Representative negatively-stained TEM images of C_{16}-($PEP_{Au}^{M\text{-}ox}$)$_2$-based fibers. These images, and others, were used for determining the fiber length distribution.

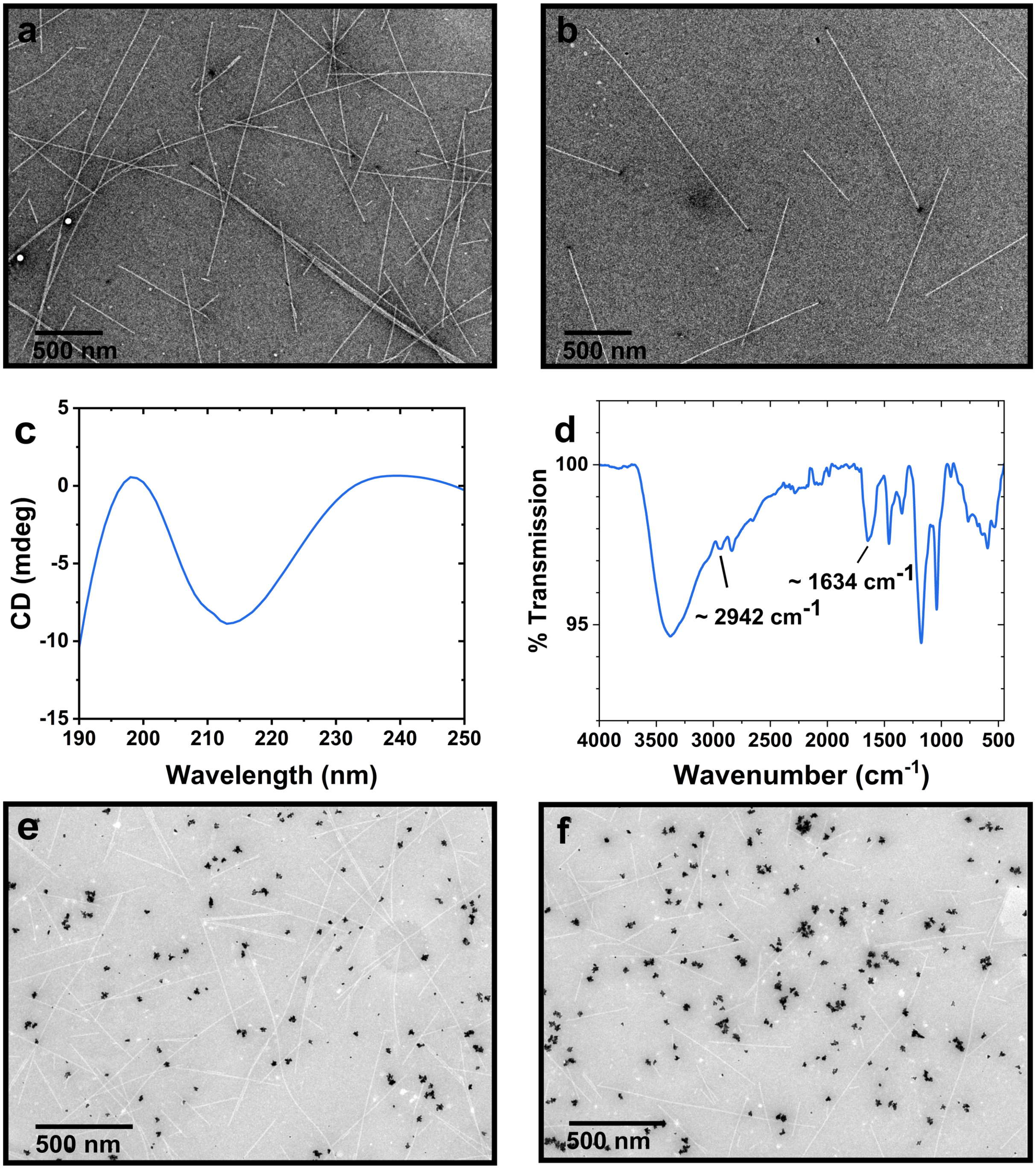

Figure S6. C_{16}-(AYSSGA)$_2$ fiber formation studies and Au NP-binding studies. (a,b) Negatively-stained TEM images of C_{16}-(AYSSGA)$_2$-based fibers. (c) CD spectrum and (d) FT-IR spectrum of fibers. (e,f) Negatively-stained TEM images of samples in which C_{16}-(AYSSGA)$_2$ was subjected to typical single helix synthesis conditions. No NP assemblies are observed, and the Au NP do not associate with the C_{16}-(AYSSGA)$_2$-based fibers.

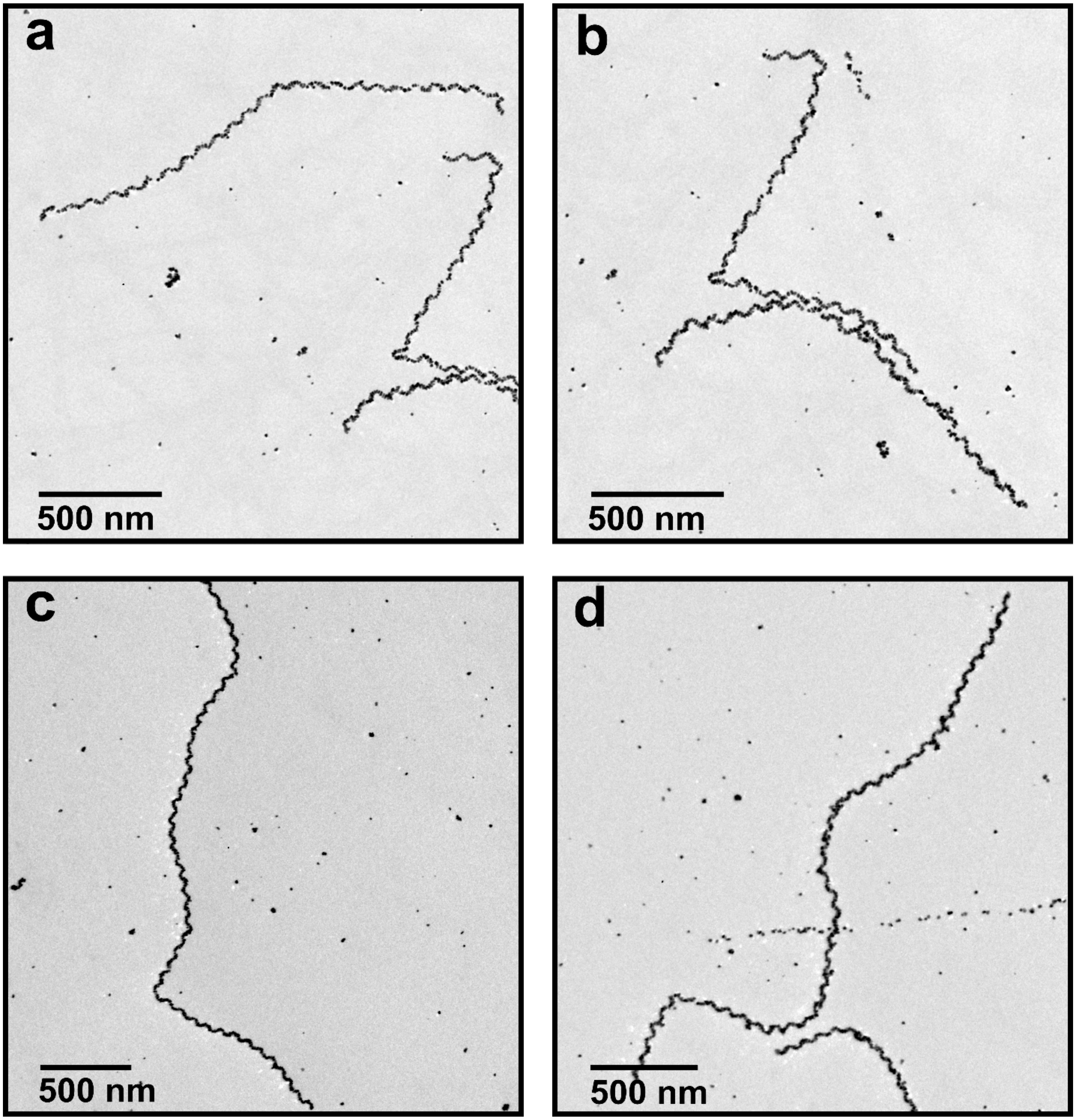

Figure S7. Additional TEM images of Au NP single helices formed using 10:1 C_{16}-($PEP_{Au}^{M\text{-}ox}$)$_2$: C_{16}-(AYSSGA)$_2$. These images, and others, were used for determining the helix length distribution.

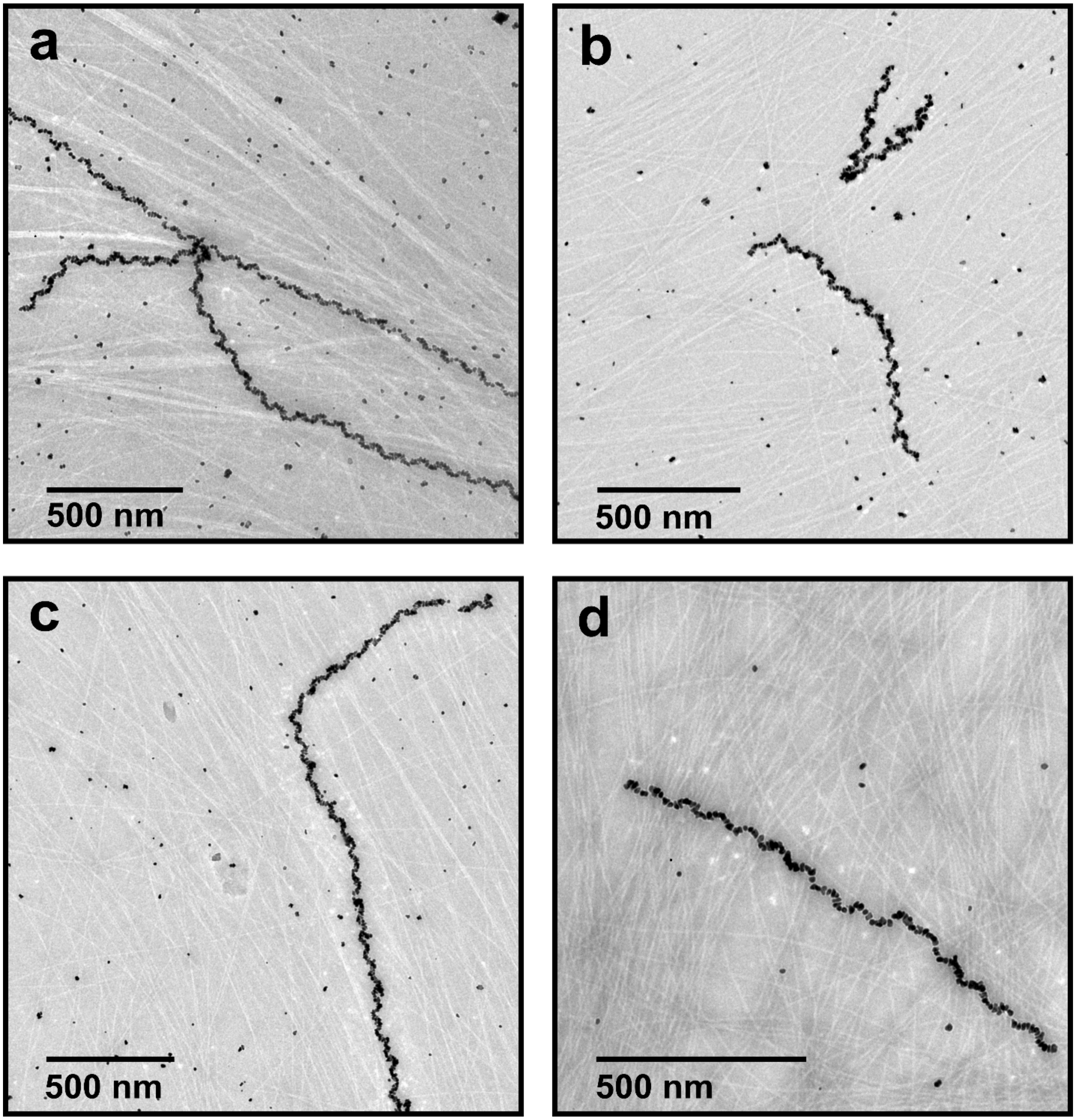

Figure S8. Negatively-stained TEM images of Au NP single helices formed using 10:1 C_{16}-$(PEP_{Au}^{M\text{-}ox})_2$: C_{16}-$(AYSSGA)_2$. These images, and others, were used for determining the fiber length distribution.

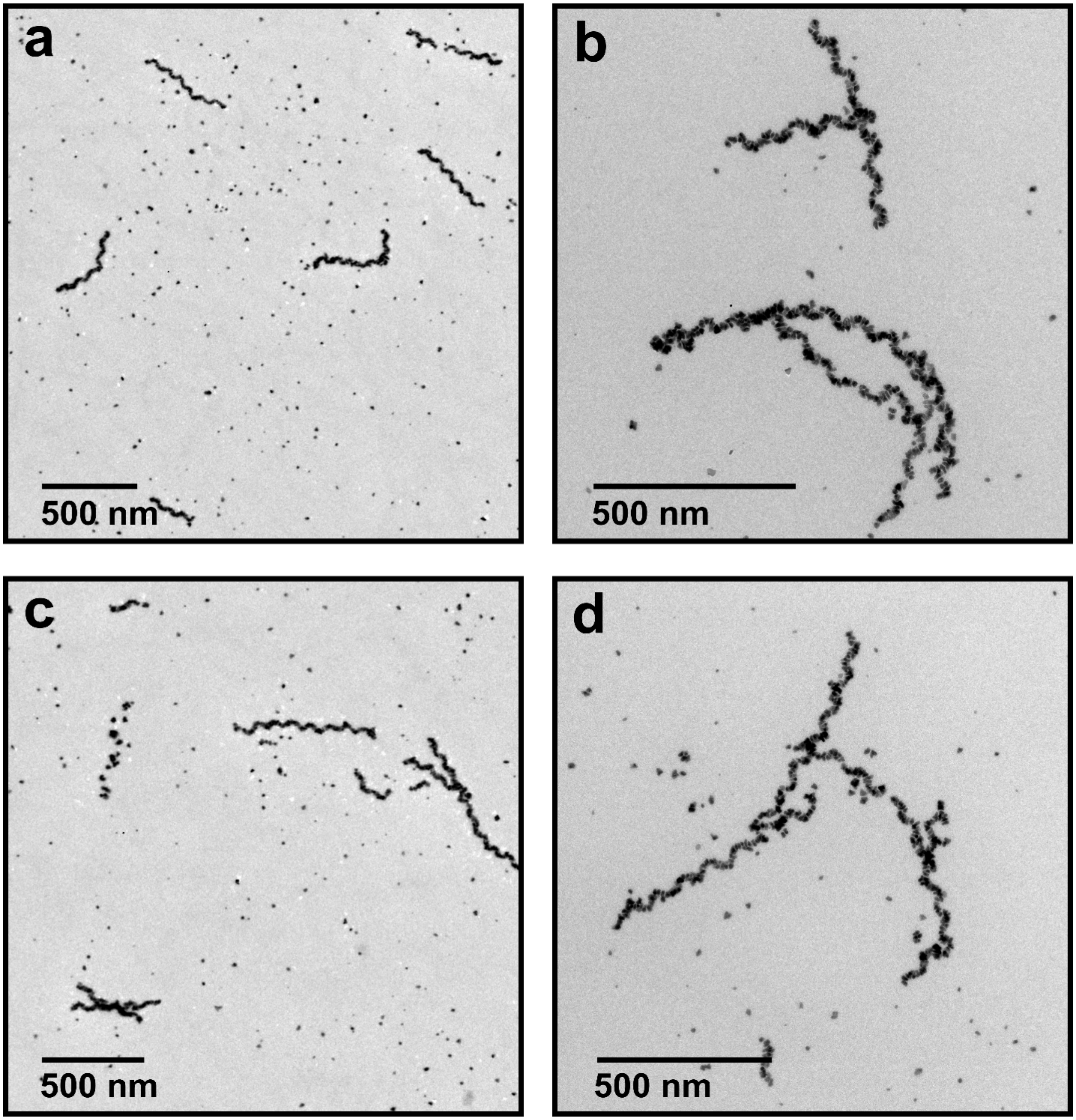

Figure S9. Additional TEM images of Au NP single helices formed using 10:5 C_{16}-($PEP_{Au}^{M\text{-}ox}$)$_2$: C_{16}-(AYSSGA)$_2$. These images, and others, were used for determining the helix length distribution.

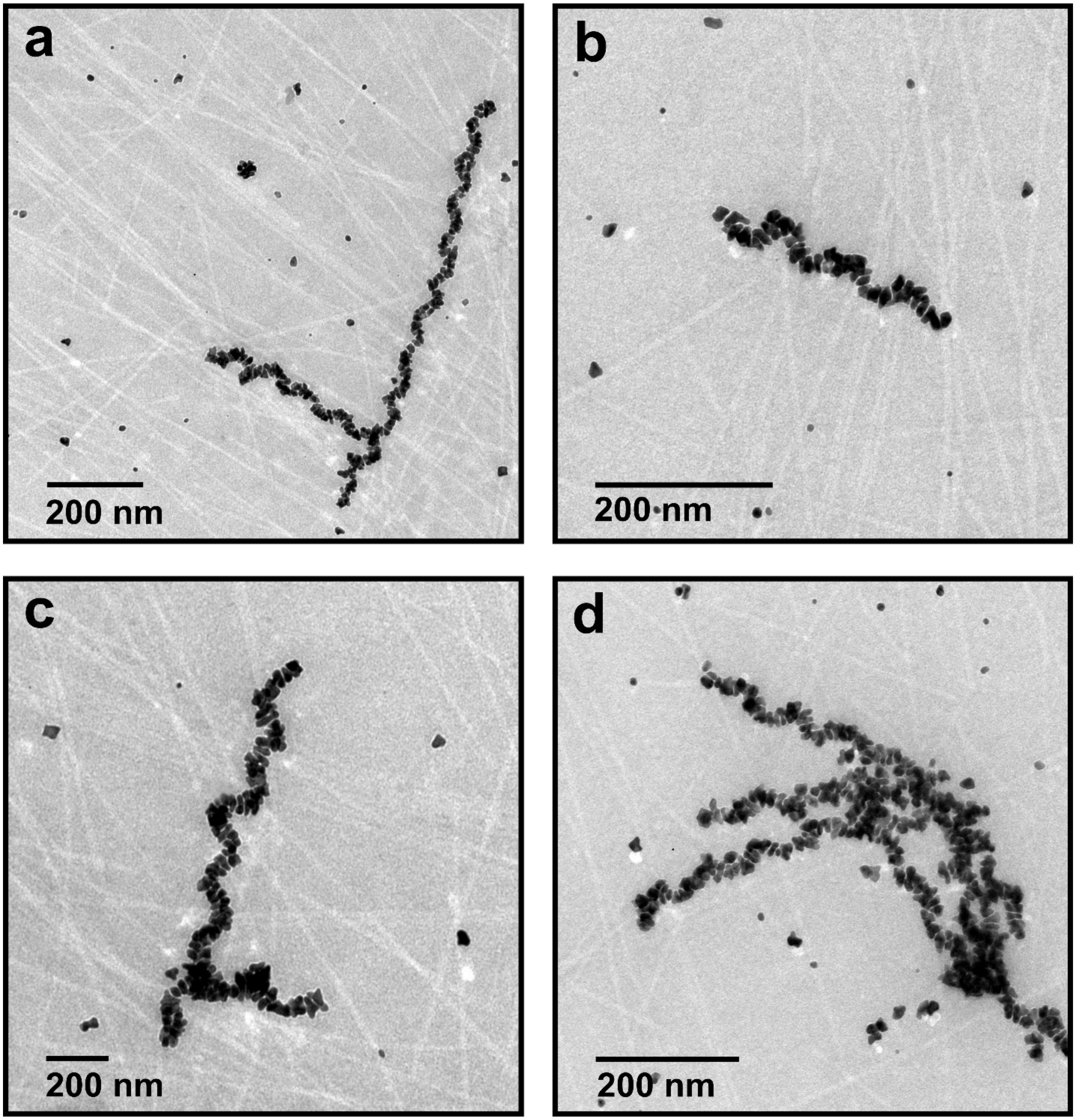

Figure S10. Negatively-stained TEM images of Au NP single helices formed using 10:5 C_{16}-($PEP_{Au}^{M\text{-}ox}$)$_2$: C_{16}-(AYSSGA)$_2$. These images, and others, were used for determining the fiber length distribution.

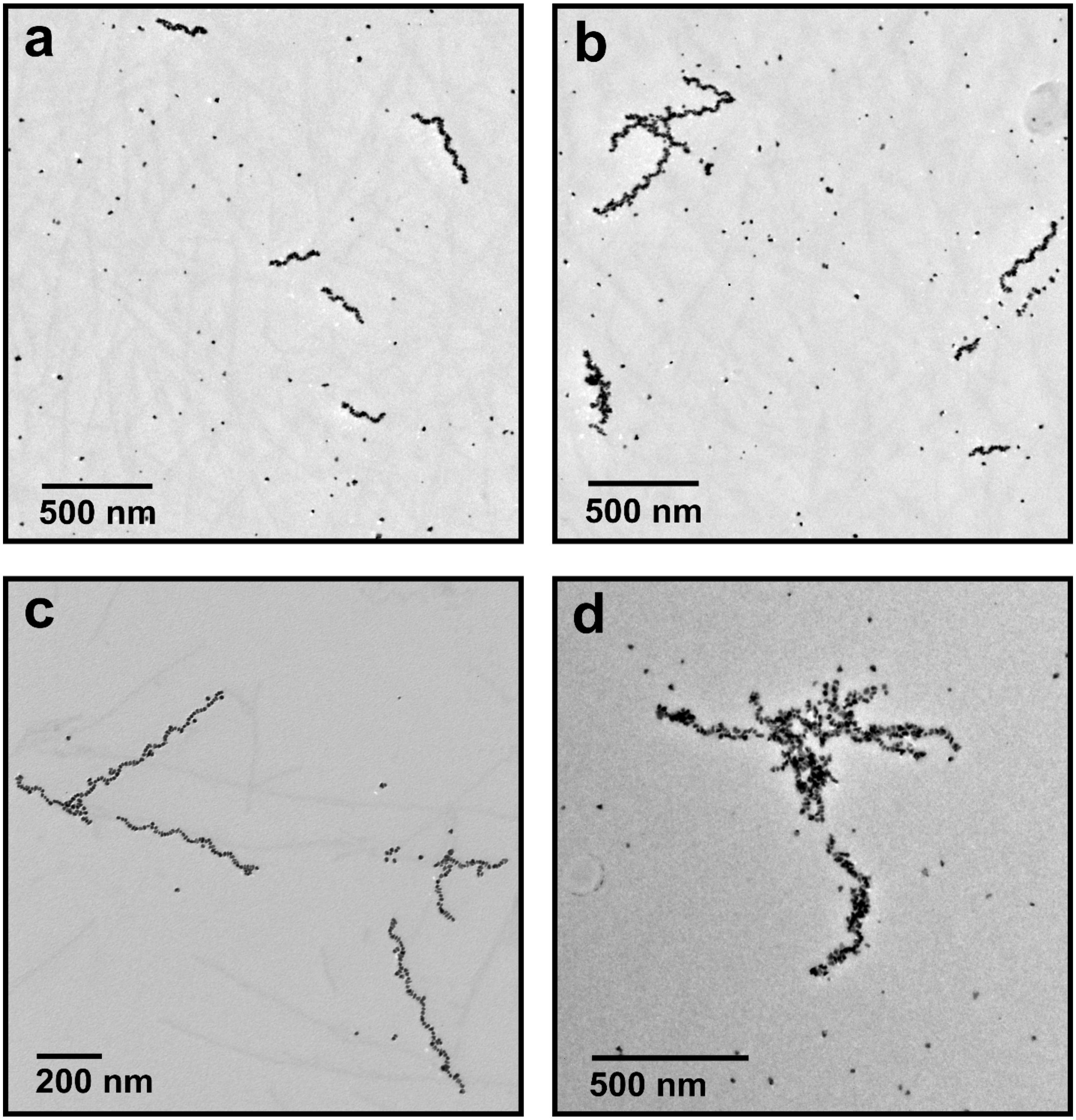

Figure S11. Additional TEM images of Au NP single helices formed using 10:10 C_{16}-(PEP_{Au}^{M-ox})$_2$: C_{16}-(AYSSGA)$_2$. These images, and others, were used for determining the helix length distribution.

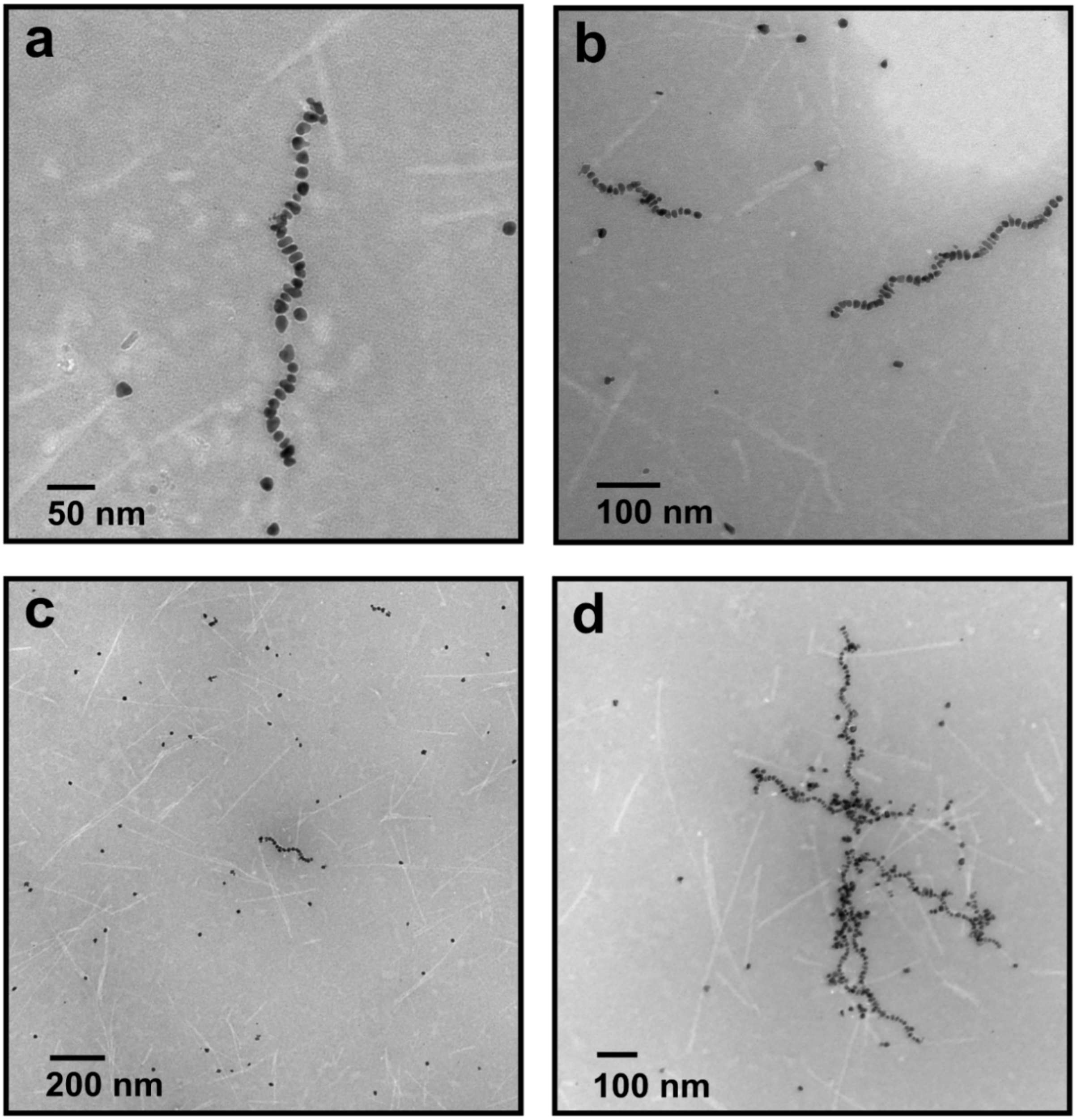

Figure S12. Negatively-stained TEM images of Au NP single helices formed using 10:10 C_{16}-$(PEP_{Au}^{M\text{-}ox})_2$: C_{16}-$(AYSSGA)_2$. These images, and others, were used for determining the fiber length distribution.

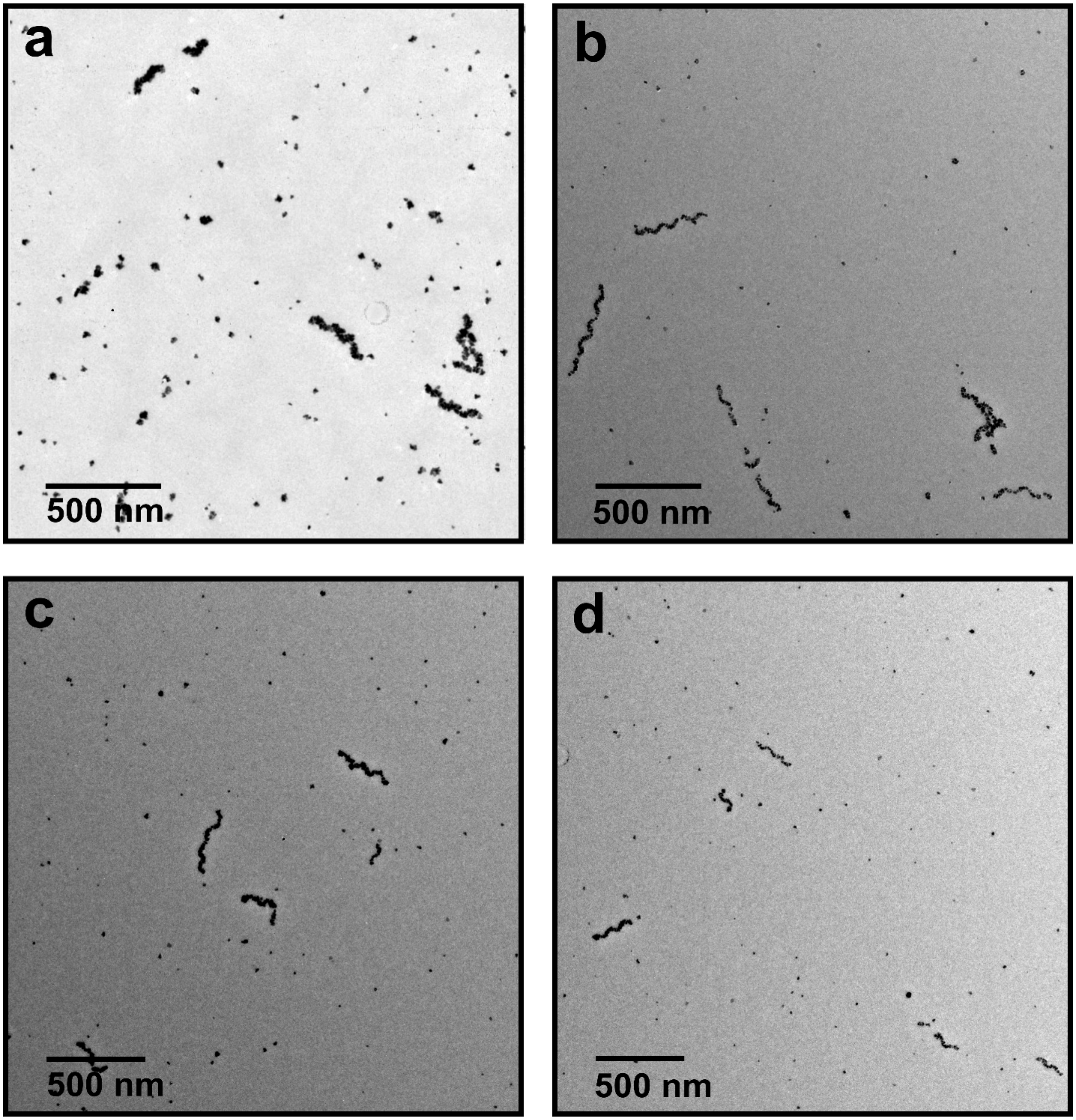

Figure S13. Additional TEM images of Au NP single helices formed using 10:15 C_{16}-(PEP_{Au}^{M-ox})$_2$: C_{16}-(AYSSGA)$_2$. These images, and others, were used for determining the helix length distribution.

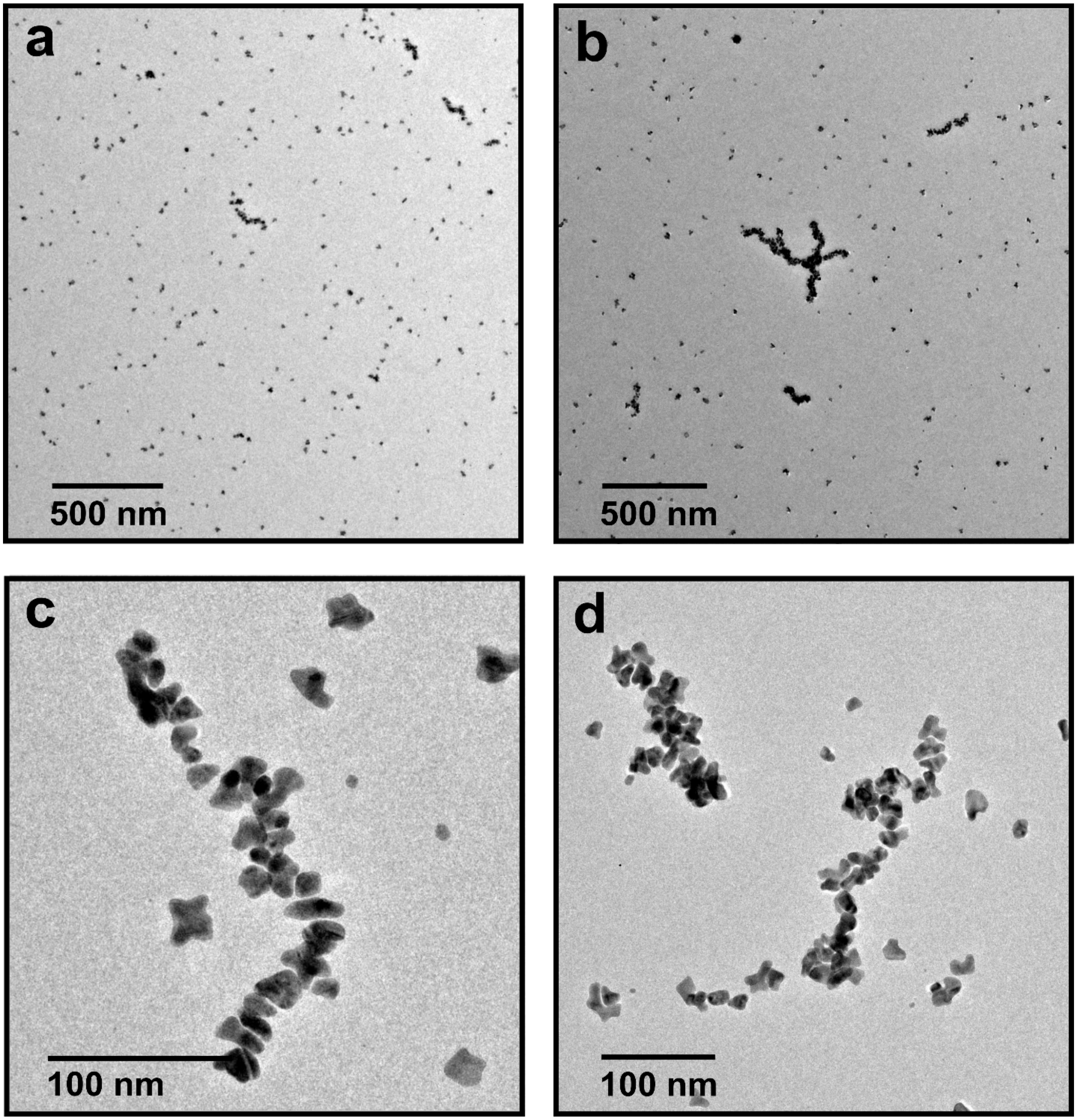

Figure S14. Representative TEM images of Au NP single helices formed using 10:25 C_{16}-($PEP_{Au}^{M\text{-}ox}$)$_2$: C_{16}-(AYSSGA)$_2$. These images, and others, were used for determining the helix length distribution.

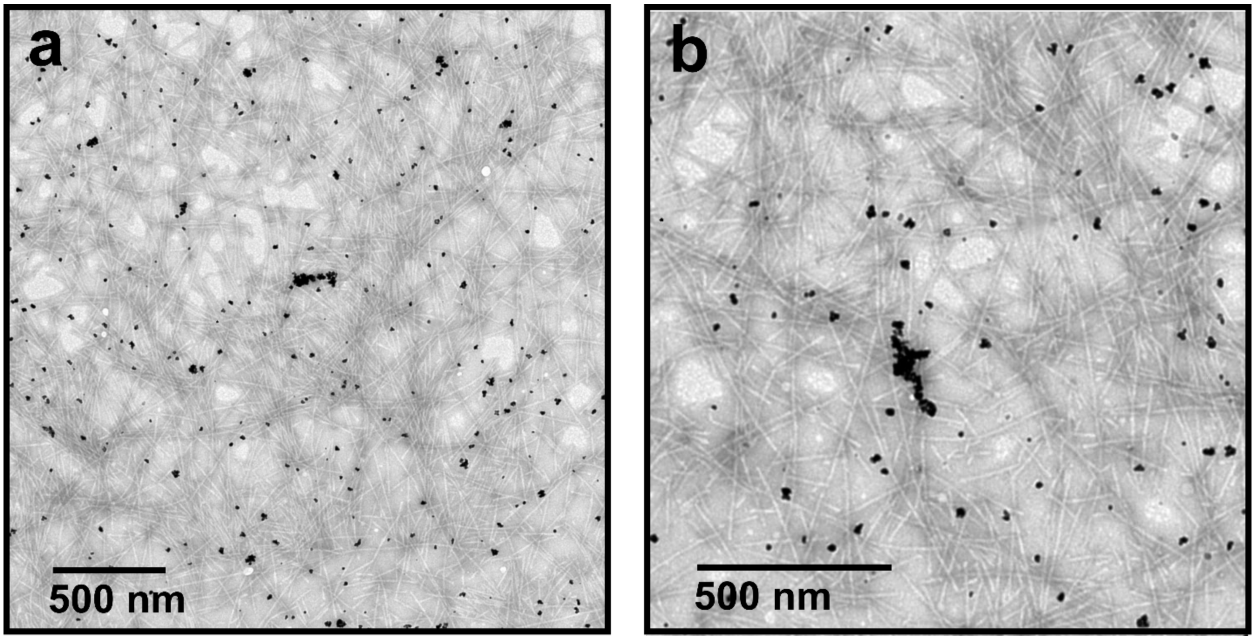

Figure S15. Negatively-stained TEM images of Au NP single helices formed using 10:25 C_{16}-$(PEP_{Au}^{M\text{-}ox})_2$: C_{16}-$(AYSSGA)_2$. These images, and others, were used for determining the fiber length distribution.

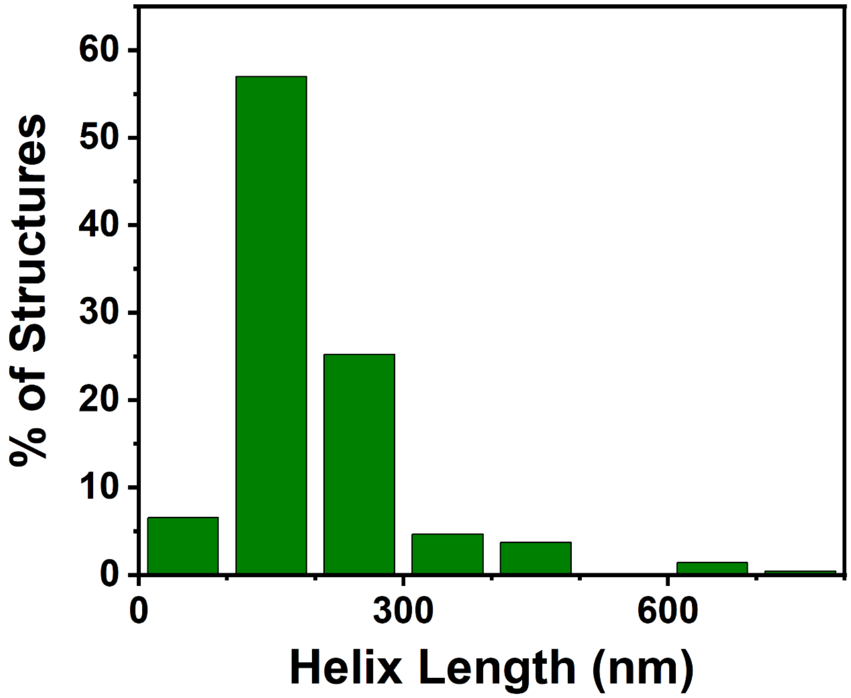

Figure S16. Helix length distribution of Au NP single helices formed using 10:25 C_{16}-$(PEP_{Au}^{M\text{-}ox})_2$: C_{16}-$(AYSSGA)_2$; measurement based on 214 counts.

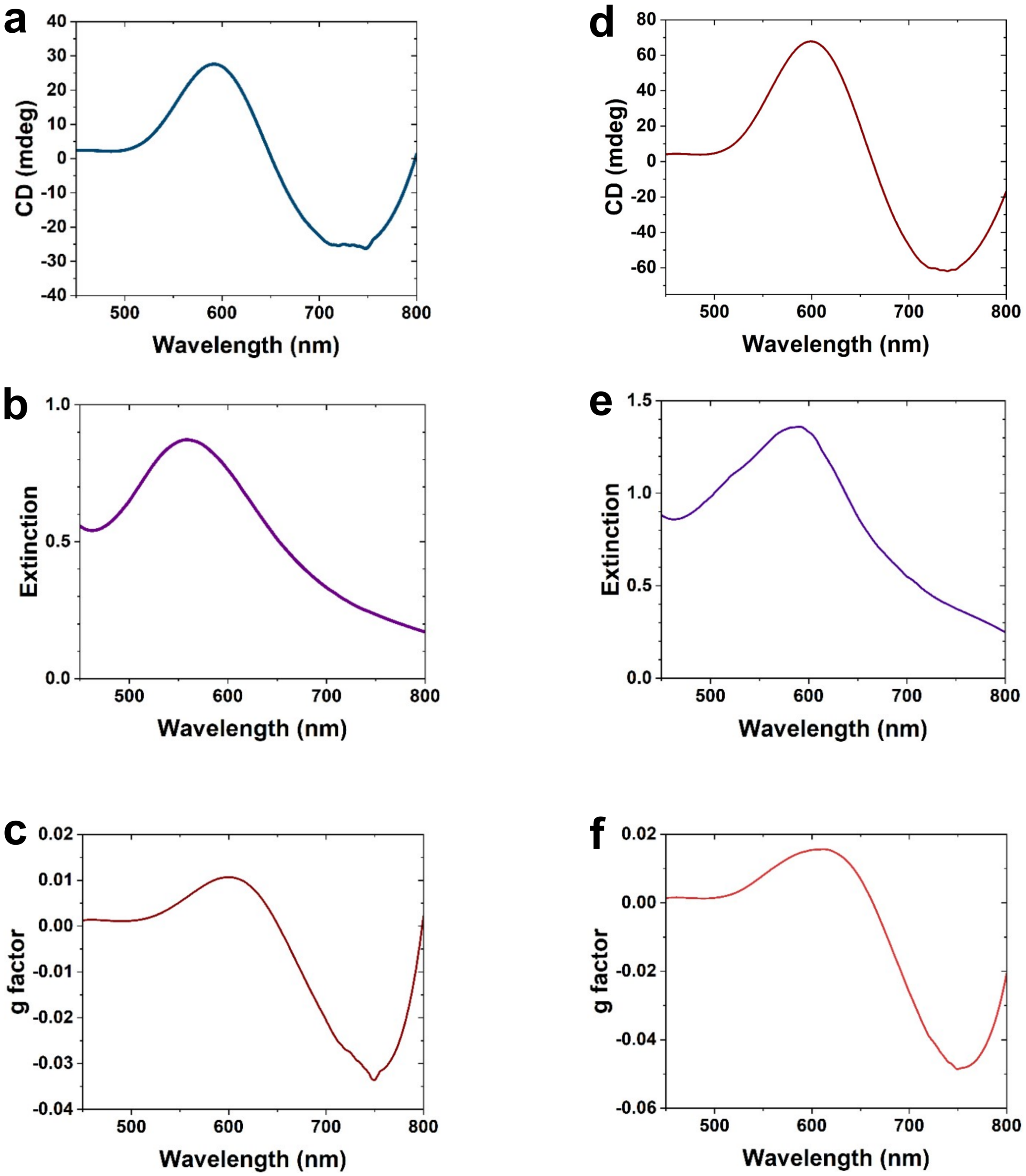

Figure S17. S18. Plasmonic chiroptical activity of Au NP single helices formed using either 10:1 or 10:5 C_{16}-($PEP_{Au}^{M\text{-}ox}$)$_2$: C_{16}-(AYSSGA)$_2$. (a), (d) CD spectra of 10:1 and 10:5; (b), (e) UV-Vis spectra of 10:1 and 10:5; (c), (f) g factor plot of 10:1 and 10:5.

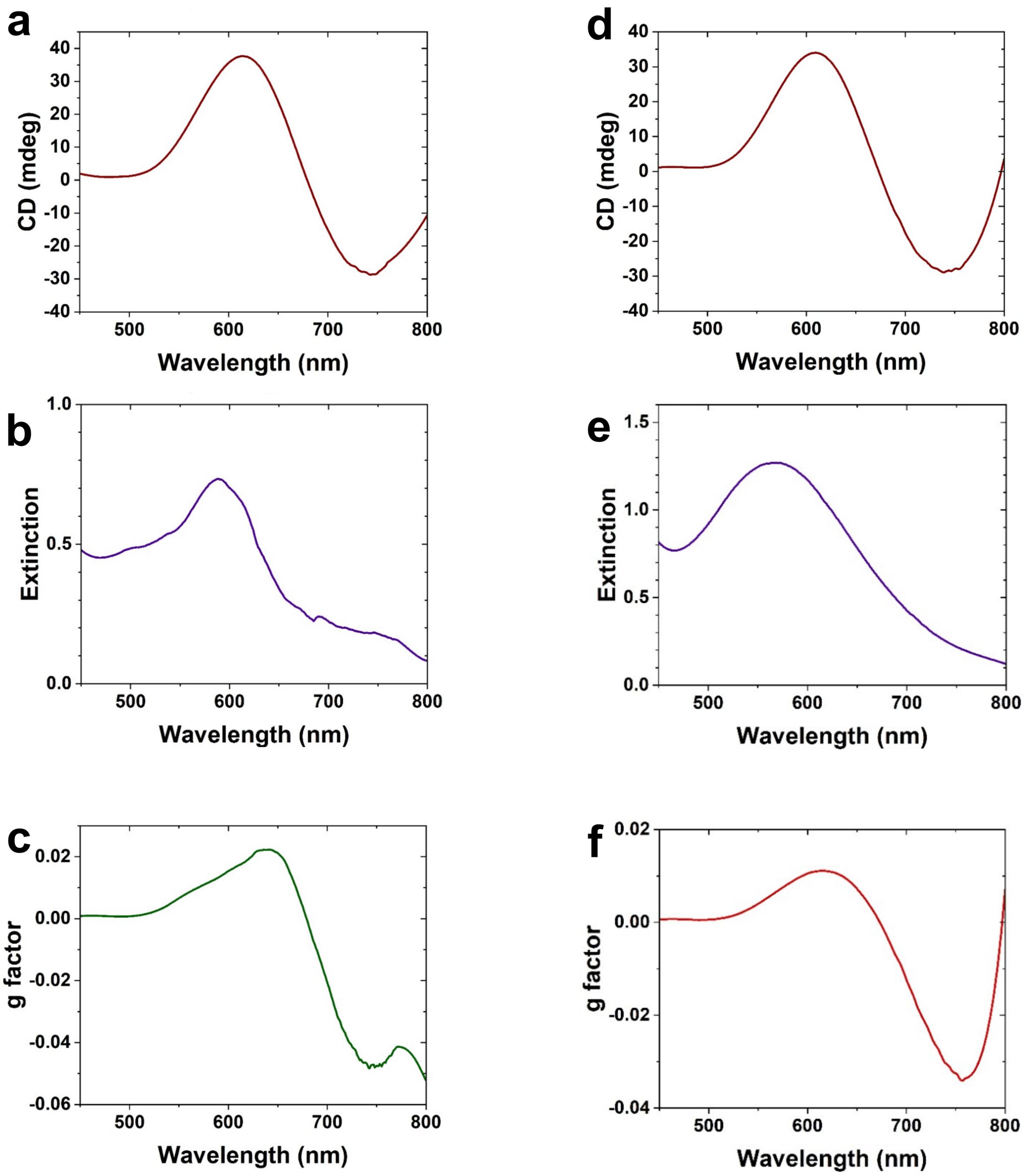

Figure S18. Plasmonic chiroptical activity of Au NP single helices formed using either 10:10 or 10:15 C_{16}-$(PEP_{Au}{}^{M\text{-}ox})_2$: C_{16}-$(AYSSGA)_2$. (a), (d) CD spectra of 10:10 and 10:15; (b), (e) UV-Vis spectra of 10:10 and 10:15; (c), (f) g factor plot of 10:10 and 10:15.

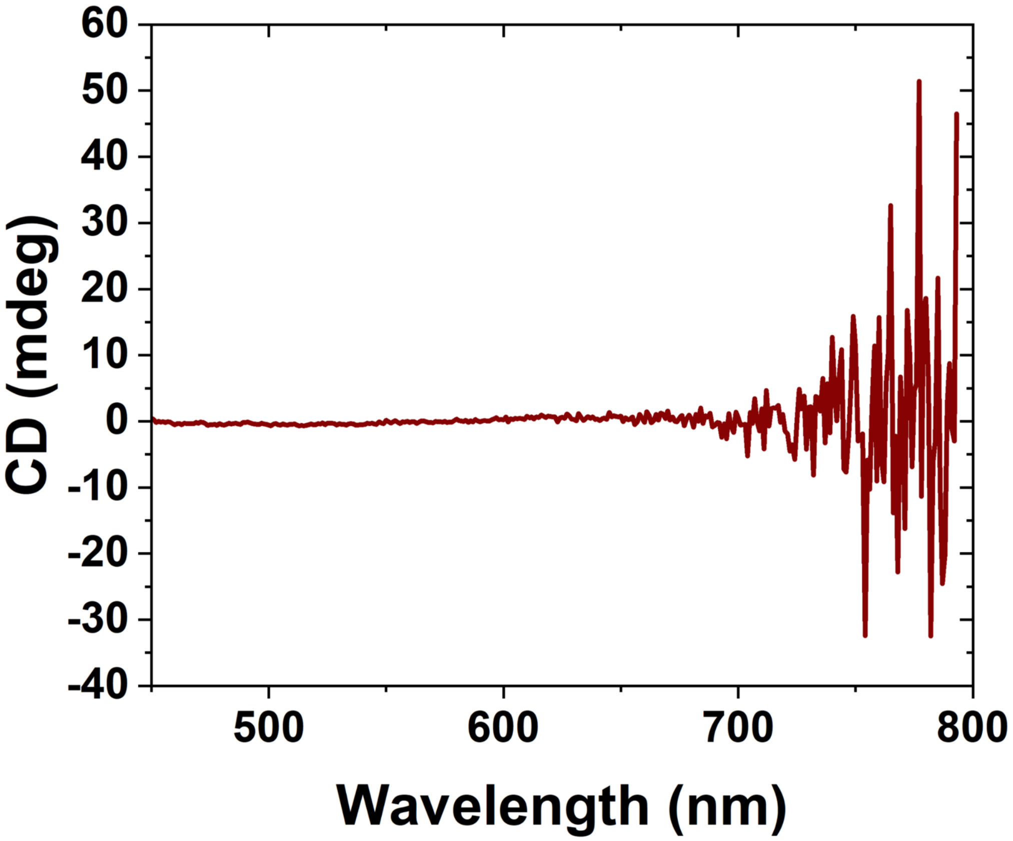

Figure S19. Plasmonic CD spectra of Au NP single helices formed using 10:25 C_{16}-($PEP_{Au}^{M\text{-}ox}$)$_2$: C_{16}-(AYSSGA)$_2$.

Sample	**Pitch length** (nm)	**Nanoparticle Width** (nm)	**Nanoparticle Length** (nm)
10:1	83 ± 12	9.4 ± 1.6	16.4 ± 3.1
10:5	82 ± 12	10.0 ± 2.0	21.1 ± 4.7
10:10	81 ± 10	11.3 ± 2.6	19.3 ± 5.1
10:15	84 ± 12	11.3 ± 1.9	17.3 ± 2.9
10:25	94 ± 18	10.8 ± 2.4	20.9 ± 4.2

Table S1. Structural parameters for single helices prepared using mixtures of C_{16}-$(PEP_{Au}^{M\text{-}ox})_2$ and C_{16}-$(AYSSGA)_2$. All measurement based on 100 counts.

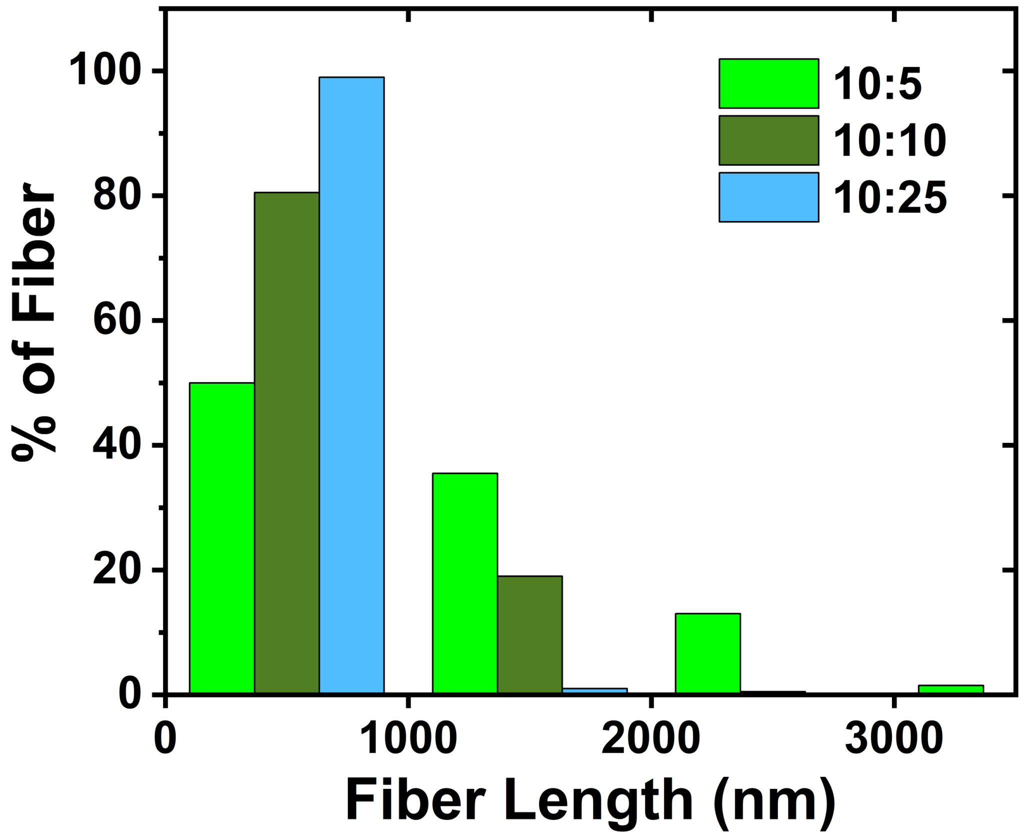

Figure S20. Length distributions of fibers prepared using mixtures of C_{16}-($PEP_{Au}^{M\text{-}ox}$)$_2$ and C_{16}-(AYSSGA)$_2$. Data based on measurements of fibers in Figures S10, S12, and S15 (150 counts for each condition).

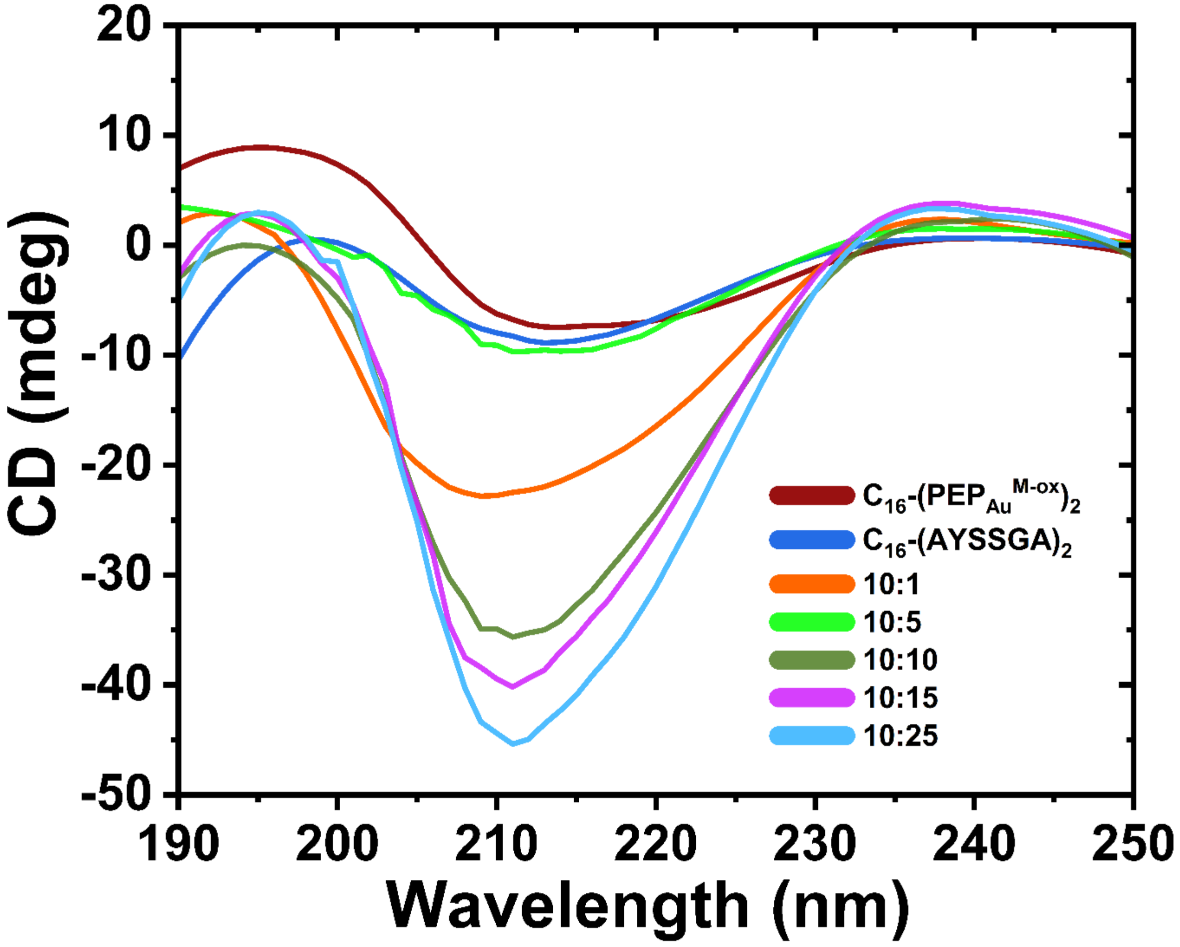

Figure S21. CD spectra of fibers prepared using mixtures of C_{16}-($PEP_{Au}^{M\text{-}ox}$)$_2$ and C_{16}-(AYSSGA)$_2$.

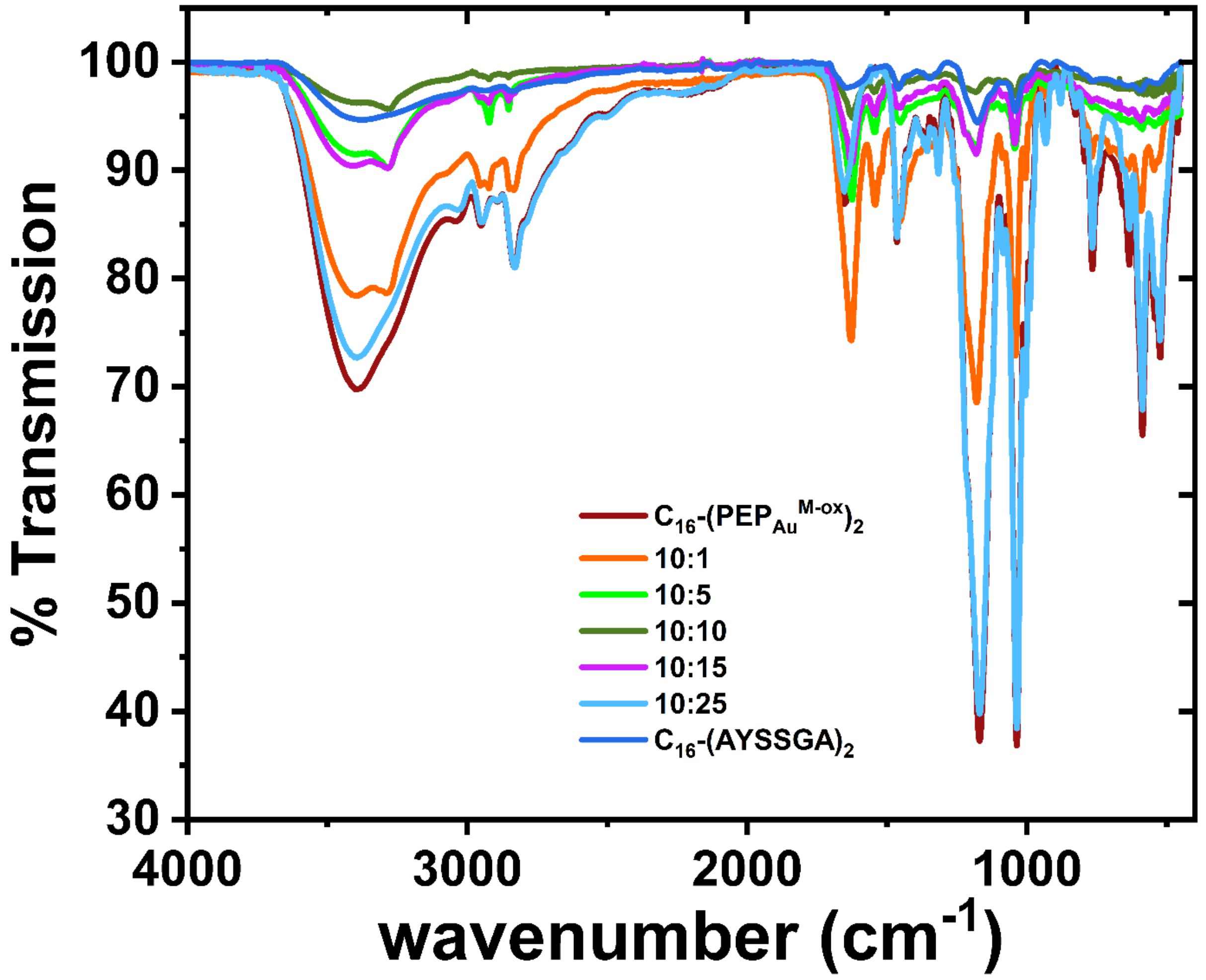

Figure S22. FTIR spectra of fibers prepared using mixtures of C_{16}-($PEP_{Au}^{M\text{-}ox}$)$_2$ and C_{16}-(AYSSGA)$_2$.

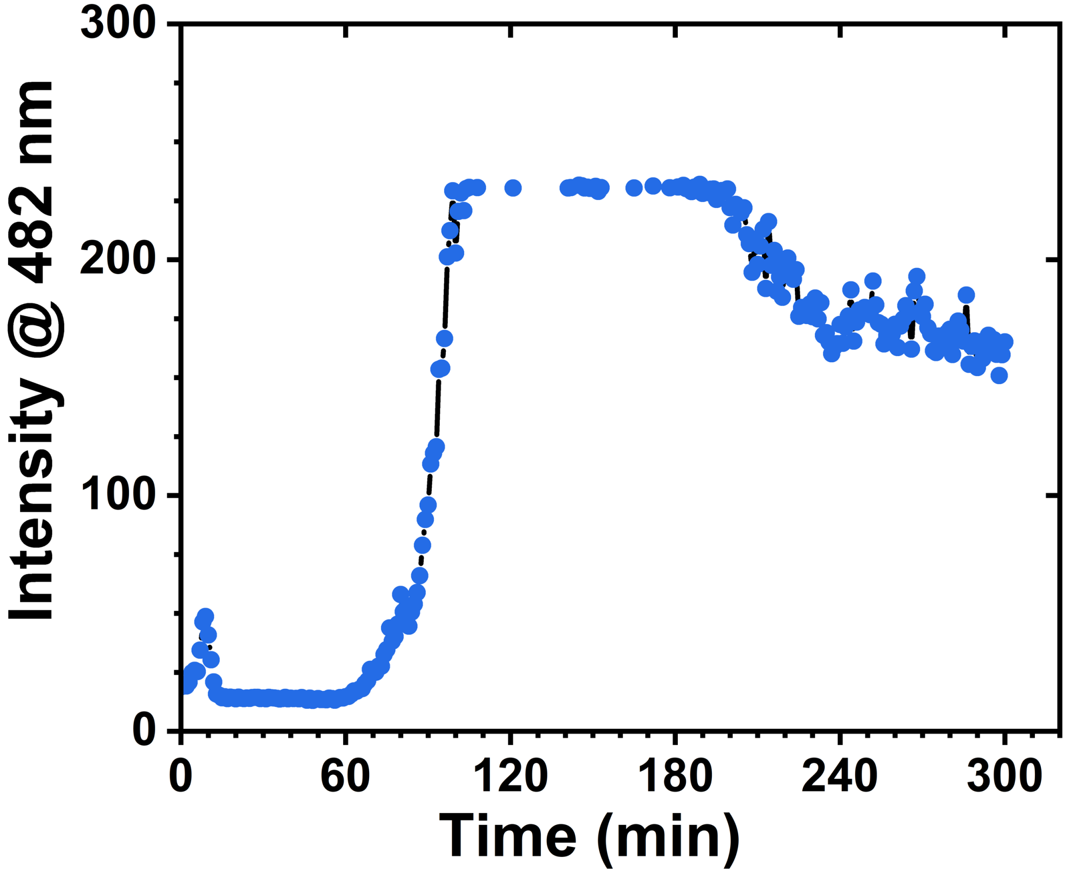

Figure S23. ThT fluorescence study of modulator assembly in 1:1 mixture of acetonitrile and H_2O.

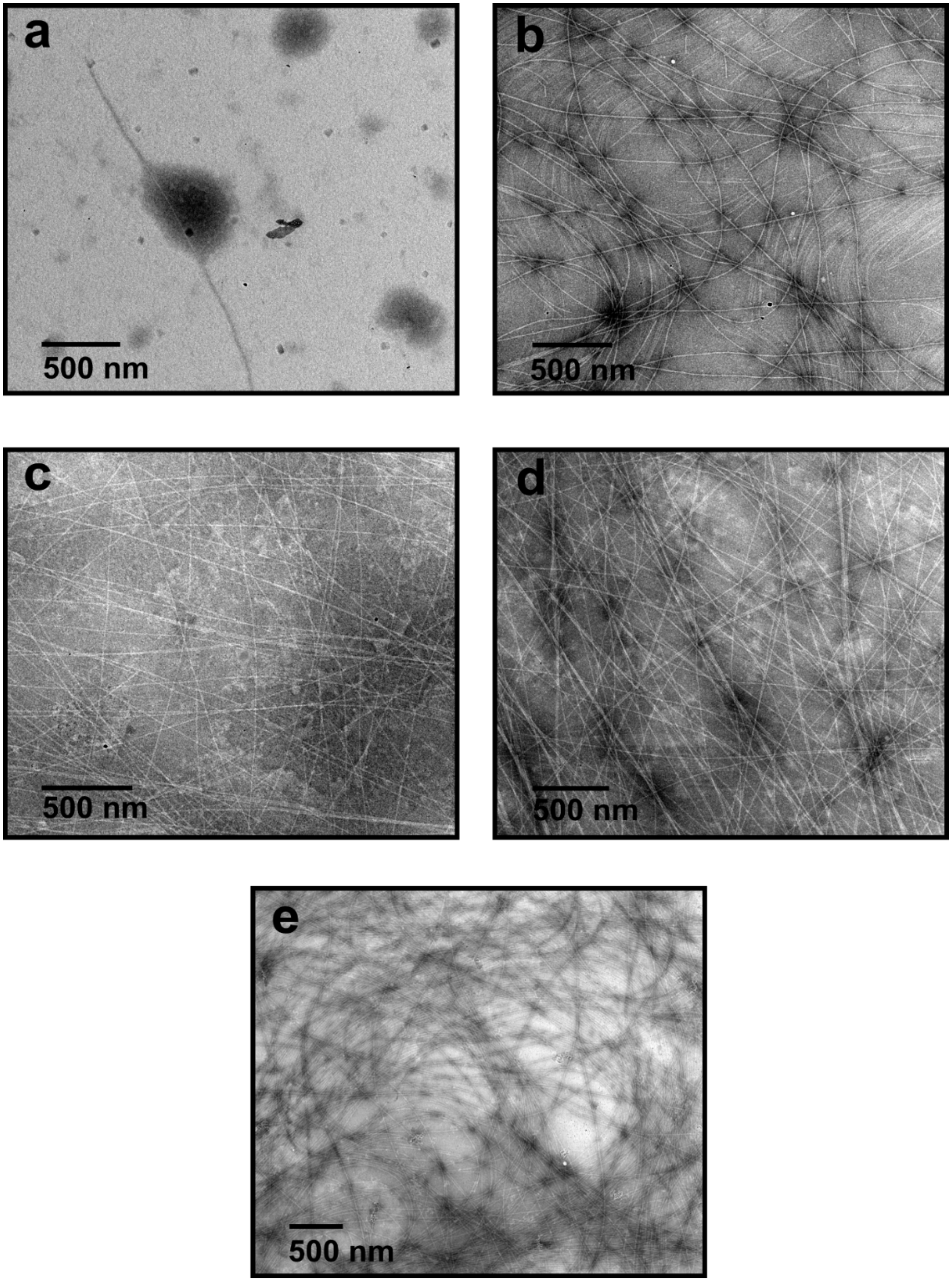

Figure S24. Negatively-stained TEM images of C_{16}-$(PEP_{Au}^{M\text{-}ox})_2$-based fibers at different assembly timepoints: (a) 0 min, (b) 1 h, (c) 2 h, (d) 3h, and (e) 18h.

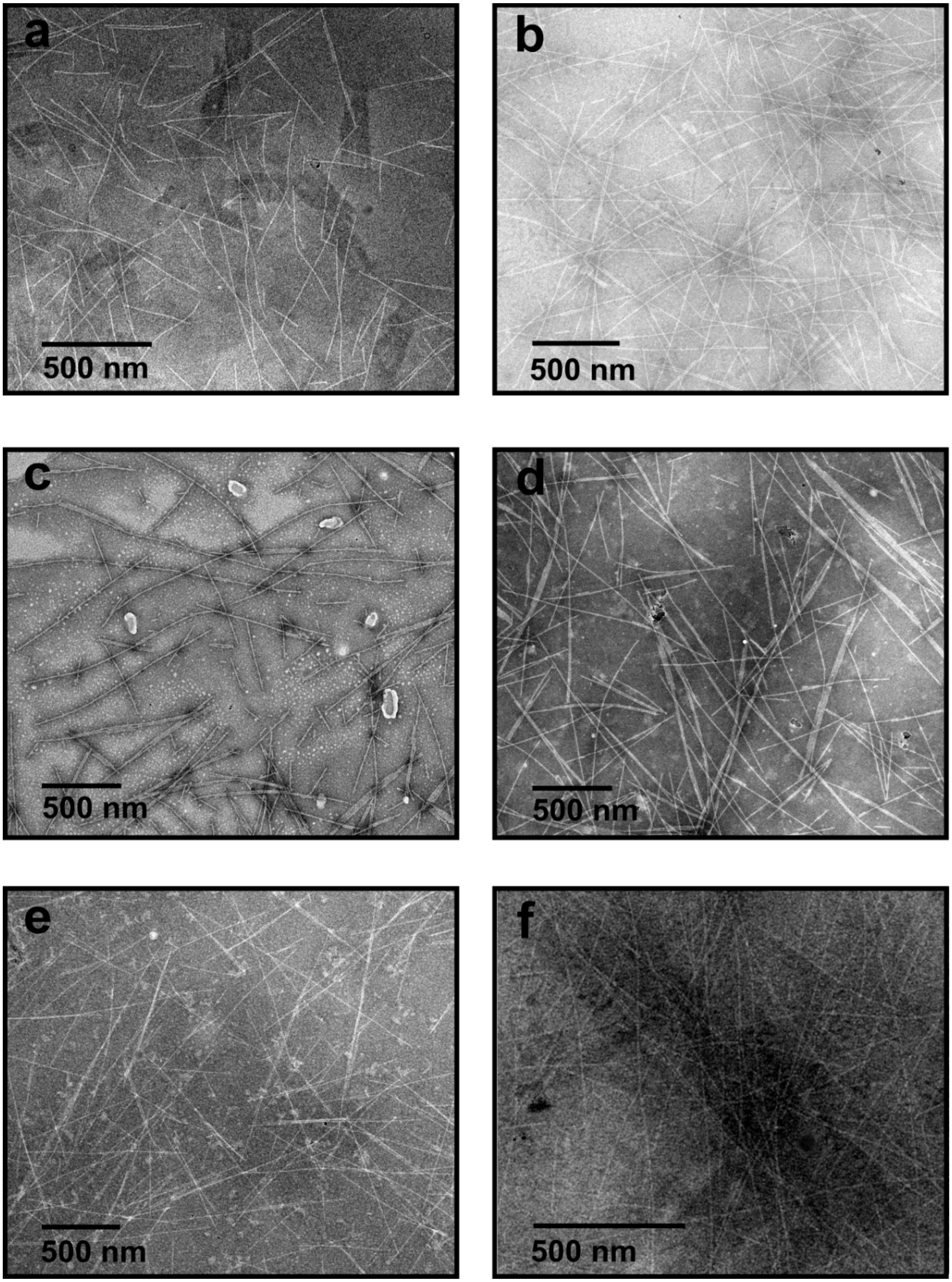

Figure S25. Negatively-stained TEM images of C_{16}-$(AYSSGA)_2$-based fibers at different assembly timepoints: (a) 0 min, (b) 30 min, (c) 2 h, (d) 3 h, (e) 4 h, and (f) 18 h.

Time	Modulator Fiber Length		
	< 1000 nm	Median (nm)	Average (nm)
0 min	~93%	~447	~503
1 h	~93%	~400	~485
3 h	~58%	~910	~999
18 h	~50%	~993	~1119

Table S2: C_{16}-$(AYSSGA)_2$-based fiber lengths at different assembly timepoints (measurements based on 150 counts).

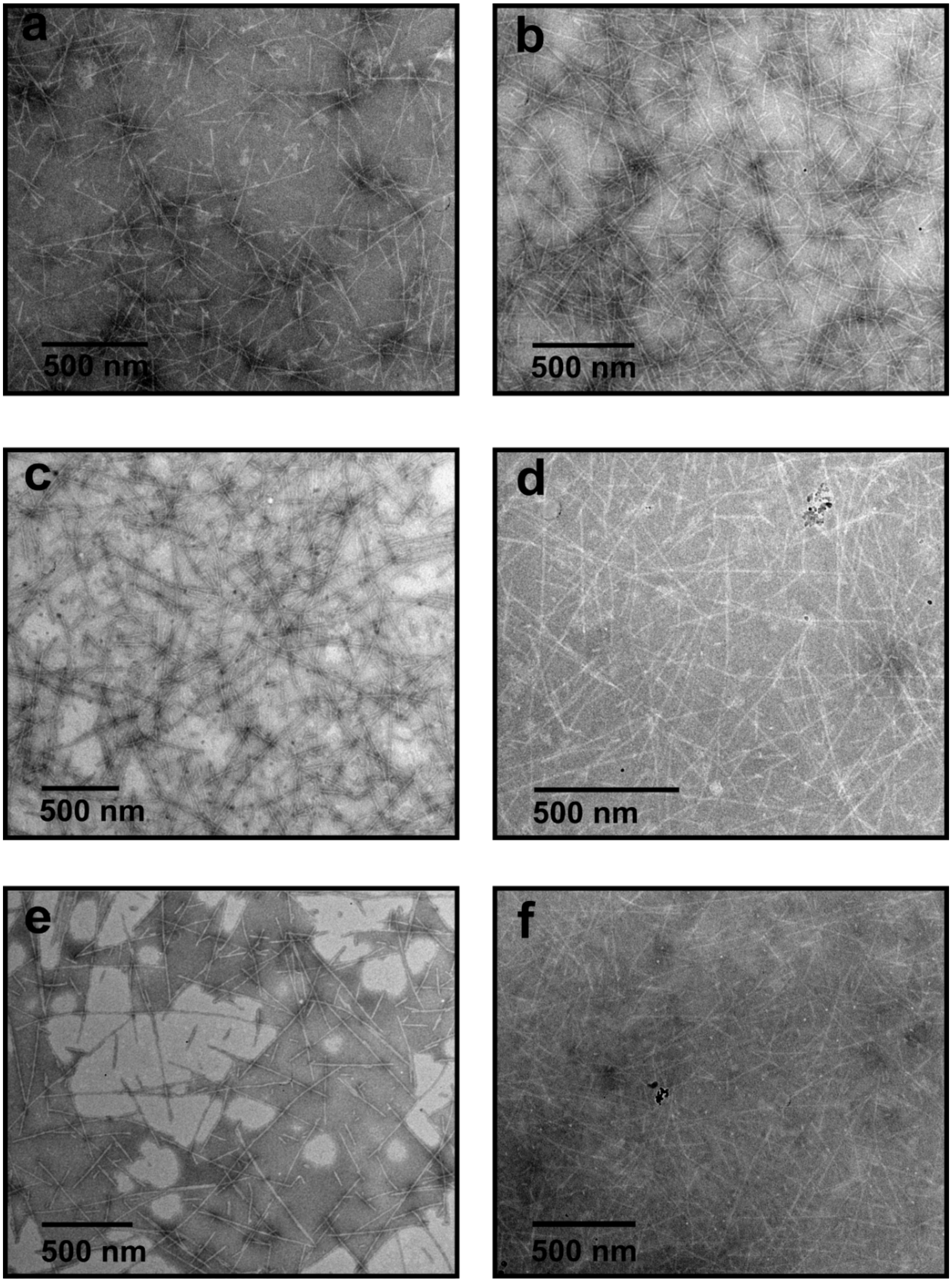

Figure S26. Negatively-stained TEM images of fibers formed from 10:10 C_{16}-$(PEP_{Au}^{M\text{-}ox})_2$: C_{16}-$(AYSSGA)_2$ at different assembly timepoints: (a) 0 min, (b) 30 min, (c) 1 h, (d) 3 h, (e) 4 h, and (f) 18 h.

Time	"10:10" Fiber Length		
	< 1000 nm	Median (nm)	Average (nm)
0 min	~100%	~259	~293
30 min	~88%	~548	~601
4 h	~74%	~720	~926
18 h	~87%	~640	~677

Table S3: Lengths of fibers formed using 10:10 C_{16}-($PEP_{Au}^{M\text{-}ox}$)$_2$: C_{16}-(AYSSGA)$_2$ at different assembly timepoints (measurement based on 150 counts).

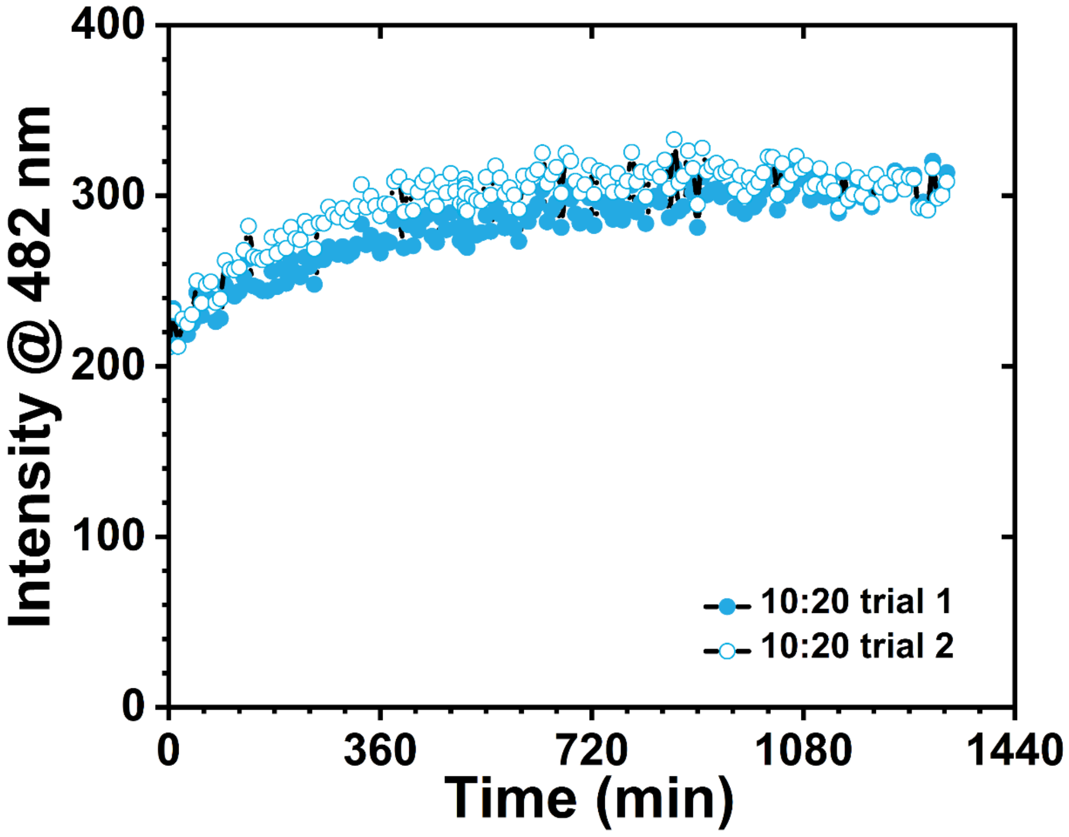

Figure S27. ThT fluorescence study of fiber assembly of 10:20 C_{16}-$(PEP_{Au}^{M\text{-}ox})_2$: C_{16}-$(AYSSGA)_2$.

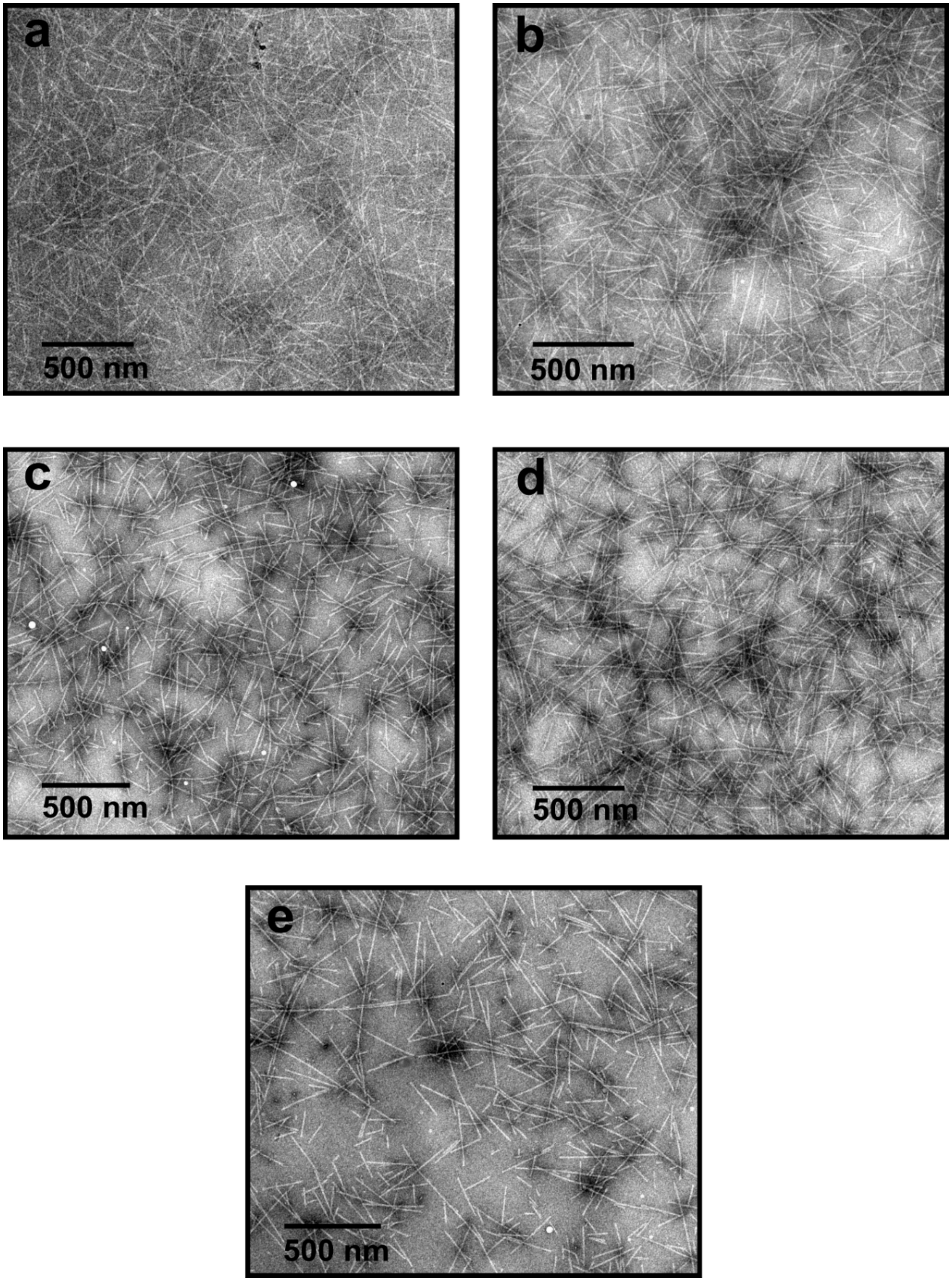

Figure S28. Negatively-stained TEM images fibers formed using 10:20 C_{16}-$(PEP_{Au}{}^{M\text{-}ox})_2$: C_{16}-$(AYSSGA)_2$ at different assembly timepoints: (a) 0 min, (b) 1 h, (c) 2 h, (d) 3 h, and (e) 48 h.

Time	"10:20" Fiber Length		
	< 1000 nm	Median (nm)	Average (nm)
0 min	~100%	~341	~388
1 h	~99%	~305	~329
3 h	~100%	~363	~390
48 h	~100%	~300	~335

Table S4: Lengths of fibers formed using 10:20 C_{16}-(PEP_{Au}^{M-ox})$_2$: C_{16}-(AYSSGA)$_2$ at different assembly timepoints (measurement based on 150 counts).
